# A pH Sensitive High-Throughput Assay for miRNA Binding of a Peptide-Aminoglycoside (PA) Library

**DOI:** 10.1371/journal.pone.0144251

**Published:** 2015-12-11

**Authors:** Derrick Watkins, Liuwei Jiang, Smita Nahar, Souvik Maiti, Dev P. Arya

**Affiliations:** 1 Laboratory of Medicinal Chemistry, Department of Chemistry, Clemson University, Clemson, South Carolina, United States of America; 2 NUBAD, LLC, Greenville, South Carolina, United States of America; 3 Academy of Scientific and Innovative Research (AcSIR), Anusandhan Bhawan, 2 Rafi Marg, New Delhi, India; 4 CSIR- Institute of Genomics and Integrative Biology, Delhi, India; National University of Singapore, SINGAPORE

## Abstract

MicroRNAs (miRNA) are small RNAs that have a regulatory role in gene expression. Because of this regulatory role, miRNAs have become a new target for therapeutic compounds. Here, we outline an approach to target specific miRNAs using a high throughput capable assay and a 215 compound peptidic-aminosugar (PA) library. Aminosugars have been shown in a number of recent reports as important lead compounds that bind miRNA. In order to screen for compounds that bind miRNA, we have developed a high throughput displacement assay using a fluorescein-neomycin conjugated molecule (F-neo) as a probe for competitive miRNA binding compounds. We have applied the F-neo assay to four different miRNA constructs and the assay is applicable to most miRNAs, at various stages of processing. The results of the screen were validated by the determination of the IC_50_ for a select group of compounds from the library. For example, we identified eight compounds that bind to hsa-miR 504 with higher affinity than the parent neomycin. From the F-neo displacement assay we found that the number of binding sites differs for each miRNA, and the binding sites appear to differ both physically and chemically, with different affinity of the compounds resulting from the size of the molecule as well as the chemical structure. Additionally, the affinity of the compounds was dependent on the identity and position of the amino acid position of conjugation and the affinity of the compounds relative to other compounds in the library was miRNA dependent with the introduction of a second amino acid.

## Introduction

Small silencing RNAs have recently been found to be fundamental regulatory molecules in the expression of protein coding genes [1]. These RNAs consist of three classes of small RNAs; small interfering RNA (siRNA), Piwi interacting RNA (piRNA) and MicroRNA (miRNA). The miRNAs are ~22 base non-protein coding RNA sequences that aid the regulation of gene expression and development by targeting mRNA for cleavage or translational suppression [2]. Since first being identified in 1993, the interest in these molecules has rapidly increased as it has been realized how fundamental these molecules are to basic biological processes [3,4].

**Fig 1:**
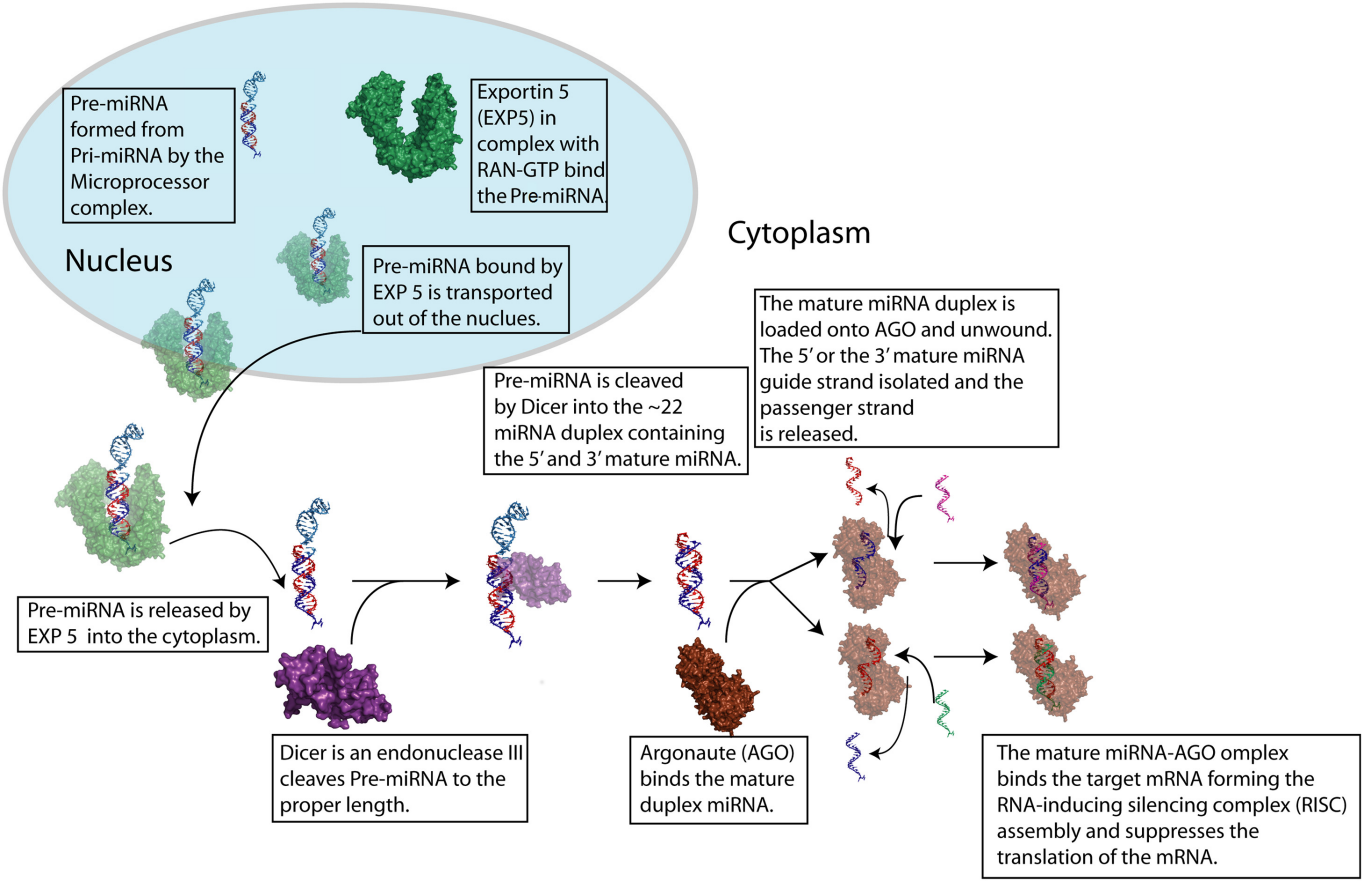
Schematic of miRNA processing. The preliminary miRNA (pre-miRNA) containing the mature 5’ (red) or 3’ (blue) strand is transported out of the nucleus by exportan5 in complex with RAN•GTP, and released into the cytoplasm. The pre-miRNA is cleaved by the endonuclease III, Dicer, to produce the mature duplex miRNA of ~22 bases per strand. The mature duplex miRNA is then loaded onto a argonaute (AGO) protein where it is unwound, the passenger strand is released, and the guide strand is arranged in the A-form conformation (modelled here as either or both the 5’ and 3’ strand is used as the guide strand). The target mRNA (lime green or magenta) is bound to the complex, resulting the suppression of its translation and protein synthesis. EXP5 (green) was modelled from PDB id:3A6P, Dicer (purple) was from PDB id:4NH5, and AGO (brown) was modelled from PDB id:4W5R.

When first transcribed by RNA polymerase II, the mature miRNA is embedded in the stem loop region of the much larger primary miRNA (pri-miRNA)[5,6]. The pri-miRNA is processed initially by the nuclear RNase III, Drosha, and the nuclear localized protein, DGCR8, to the ~65 nucleotide pre-miRNA[7,8]. Following the initial processing of the pri-miRNA the resulting pre-miRNA is escorted out of the nucleus by the protein exportin 5 (EXP5) and released into the cytosol (Fig 1)[9]. A final cleavage of the pre-miRNA in the cytoplasm by the endonuclease III, Dicer, results in a duplex RNA containing the mature miRNA[10]. Mature miRNA binding to target mRNA is facilitated by the argonaute class of proteins, forming the RNA-induced silencing complex (RISC) assembly. Specificity of the RISC arises from nucleotides 2–7 of the miRNA, known as the “seed” region, which forms Watson-Crick pairs with the target mRNA[11].

Regulation of mRNA translation by miRNA is likely a common mechanism in that more than 60% of the genes in humans contain one or more conserved miRNA binding site(s). [12] Additionally, errors in the regulation or function of miRNAs lead to developmental disorders, cancer, and other human diseases. [13,14] With the established importance of miRNAs, identifying compounds that bind to these small RNA sequences could be useful for the study of miRNA function and as potentially important therapeutic compounds for the treatment of diseases related to miRNAs.

Aminoglycosides bind to a variety of RNA,[15,16] DNA[17–20] hybrid DNA•RNA[21,22] molecules including the ribosomal A-site,[23,24] DNA G-quadruplex motifs,[25,25–29] and triplex DNA,[30–36] as well as HIV TAR RNA[37,38]. Importantly, it has recently been demonstrated that aminoglycosides bind with high affinity to pre-miRNA and may act as potential inhibitors of Drosher or Dicer processing and downstream functions of mature miRNA [39–41]. At least one binding site of the aminoglycosides is likely to be within or in close proximity to the mature miRNA region [19]. Compounds that bind to the mature miRNA have the greatest potential to exploit the specificity of miRNAs. Additionally, aminoglycosides are known to bind within the groove(s) of nucleic acid structures where they have high potential for specific binding [42]. New approaches to aminoglycoside based leads that could bind with high affinity to different RNAs would therefore greatly advance our capabilities of miRNA recognition.

**Fig 2:**
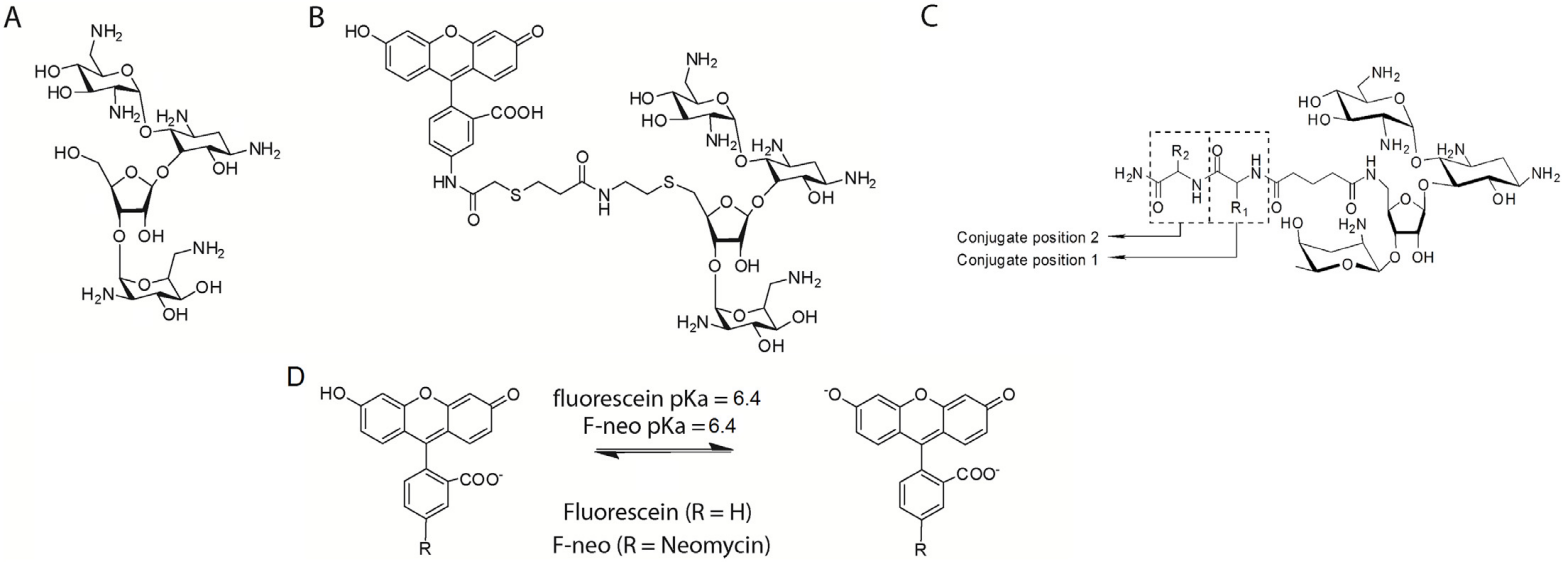
A) The chemical structures of neomycin, which was used as the positive control in the assay, B) the F-neo probe, C) the neomycin-amino acid conjugate, and D) the equilibrium between the monanion and dianion fluorescein and F-neo.

Here, we report a fluorescent displacement assay using the fluorescein-neomycin conjugated probe (F-neo, Fig 2) for the screening of compounds that bind to mature and pre-miRNA with high affinity. We use the displacement of the F-neo to evaluate the binding affinity of the PA library. The F-neo probe has been previously used to determine the affinity of compound libraries for the ribosomal A-site, and a complementary probe, a thiazole orange-neomycin conjugate, has been used in an assay for compounds that bind to the human telomeric G-quadruplex [24,26,43]. The assay can be used to fluorescently screen ligand binding to miRNA, and as opposed to intercalating probes, has the added advantage of not altering the structure of the miRNA.

The F-neo displacement assay is used to demonstrate the utility of a library of peptidic-aminoglycosides (PAs) that increases the specificity of aminoglycosides for miRNAs (Fig 2). Previously, we have shown that such amino acid-neomycin conjugates can be efficiently synthesized and can significantly alter the binding affinity of neomycin for A-site rRNA [43]. From our screening and binding analysis, we demonstrate here that miRNAs contain structural differences that can be used for specific miRNA targeting by small molecules. The binding site of miRNA for neomycin appears to differ both physically and chemically between different miRNAs. The screening and analysis of the PA library rapidly identifies the structural properties of PAs that may be used to specifically bind a selected miRNA.

## Materials and Methods

The library of 20 mature miRNAs was purchased from Dharmacon^TM^ in a 96 well plate format containing 1 nmole of 20 miRNAs. The plate was centrifuged at 1000 x g for 15 minutes prior to the removal of the foil covering. 100 μl of buffer (10 mM sodium phosphate (pH 6.5), 25 mM KCl, 0.05 mM EDTA) was added to each well, and the miRNA was suspended by pipetting to give a 1 μM final stock concentration of each mature miRNA. Both strands of each mature miRNA duplex hsa-miR 142 (5’-CAUAAAGUAGAAAGCACUACUUU-3’)•(5’-AGUAGUGCUUUCUACUUUAUGUU-3’), hsa-miR 335 (5’-UCAAGAGCAAUAACGAAAAAUGUUU-3’)•(5’-ACAUUUUUCGUUAUUGCUCUUGAUU-3’) and hsa-miR 504 (5’-AGACCCUGGUCUGCACUCUAUCUU-3’)•(5’-GAUAGAGUGCAGACCAGGGUCUUU-3’) selected from the miRNA library screen were obtained deprotected from Dharmacon^TM^ with UU overhangs, and annealed in buffer (25 mM KCl, 10 mM sodium phosphate, 0.05 mM EDTA at pH 6.5) before use. The full length 83 base sequence of the pre-hsa-miR 504 (5’-GCUGCUGUUGGGAGACCCUGGUCUGCACUCUAUCUGUAUUCUUACUGAAGGGAGUGCAGGGCAGGGUUUCCCAUACAGAGGGC-3’) was obtained deprotected and HPLC purified from Dharmacon^TM^ and suspended in buffer (25 mM KCl, 10 mM sodium phosphate, 0.05 mM EDTA at pH 6.5).

The peptide conjugated aminoglycoside compound library and F-neo were synthesized as previously described [43], and neomycin was obtained as a solid sulfate from Fisher Scientific. All absorbance experiments were performed in 25 mM KCl, 10 mM sodium phosphate, 0.05 mM EDTA, and the pH was adjusted as indicated using dilute HCl or NaOH as appropriate, in order to perform pH titrations (see “The quenching of F-neo upon binding miRNA is a result of a shift in the pK_a_ of fluorescein” in the “Results” section. Absorbance scans were performed on in 96-well Costar transparent plates using a Tecan Infinite M-1000 plate reader and were scanned from 400 nm to 600 nm. The pK_a_ was determined from absorbance measurements taken at 490 nm at 1 μM F-neo in the presence and absence of 1 μM mature hsa-miR 504.

All binding experiments were performed in 25 mM KCl, 10 mM sodium phosphate, 0.05 mM EDTA at pH 6.5. Fluorescence experiments were performed in a 96-well black Greiner plate and measurements were taken in a Tecan Infinite M-1000Pro plate reader with an excitation wavelength of 485 nm and an emission of 525 nm.

The mature miRNA library was screened for F-neo binding by combining 20 μl of the stock miRNA with 180 μl F-neo solution in buffer to give a final concentration of 100 nM F-neo to 100 nM miRNA. The *E*. *coli* model of the ribosomal A-site (5’-GGCGUCACACCUUCGGGUAAGUCGCC-3’) was included as a positive control and a solution containing only 100 nM F-neo was used as a negative control. Each miRNA, positive control, and negative control was screened in duplicate. The difference in the fluorescence between the F-neo only and the F-neo with the miRNA (ΔF) was determined for each experiment and the average and standard deviations were determined for each miRNA.

The relative K_D_’s of miRNA for F-neo were determined by the titration of each miRNA into F-neo. The mature hsa-miR 142, hsa-miR 335, hsa-miR 504 and the pre-hsa-miR 504 were titrated as indicated into 100 nM F-neo in buffer. The K_D_ for the binding of F-neo to each miRNA was determined by fitting the data to Eq 1 using Kaleidagraph as previously described [44].

$$F=F_{0}+(F_{b}-F_{0}/\left(2\left[Fneo\right]\right)(\left[\Psi \right]+\left[Fneo\right]+1/K_{a}-\left(\left[\Psi \right]+\left[Fneo\right]+1/K_{a}\right)2-4\left(\left[\Psi \right]\left[F-neo\right]\right)1/2$$ (1)

where F_0_ is the fluorescence in the absence of the miRNA and F_b_ is the fluorescence of the fully bound F-neo, and Ψ is the concentration of binding sites.

The IC_50_ was for neomycin was determined by the titration of neomycin into 100 nM F-neo. The neomycin was titrated into 100 nM F-neo and 100 nM hsa-miR 142, 100 nM hsa-miR 335, 50 nM hsa-miR 504, or 16.7 nM pre-hsa-miR 504. The IC_50_ was determined as the point of 50% displacement of F-neo from the miRNA from a sigmoidal fit using OriginPro 2015 graphing software.

The Z’-factor was determined using 48 wells of the positive control (F-neo + miRNA + neomycin) and 48 wells of the negative control (F-neo + miRNA) using Eq 2.

$$Z’-factor\;=\;1-3\left(\sigma_{p}+\sigma_{n}\right)/\left|\mu_{p}-\mu_{n}\right|$$ (2)

Where μ_p_ is the average of the positive control, σ_p_ is the standard deviation of the positive control, μ_n_ is the average of the negative control and σ_n_ is the standard deviation of the negative control. The Z’ factor for mature and pre-hsa-miR 504 was optimized using 300 nM neomycin to 100 nM F-neo and 50 nM mature hsa-miR-504 or 16.7 nM pre-hsa-miR 504. The Z’ factor for mature hsa-miR 142 and 335 were optimized at 500 nM neomycin to 100 nM F-neo and 100 nM miRNA.

The PA library was screened for binding the mature miRNA by the F-neo displacement assay by the addition of a single concentration of compound into a fixed concentration of miRNA and F-neo in buffer with duplicates for each compound. Each compound was added at 300 nM to 50 nM miRNA 504 and 100 nM F-neo; each compound at 300 nM was added to 16.7 nM pre-miR504 and 100 nM F-neo; and each compound at 500 nM was added to 100 nM hsa-miR 142 or hsa-miR 335 and 100 nM F-neo. Neomycin was included on each plate in duplicate and all compounds were normalized to the binding of neomycin on that plate.

### Cell Culture

MCF-7 cells were purchased from European Collection of Cell Cultures (ECACC). The cell line was maintained in Dulbecco’s Modified Eagle’s Medium (DMEM) with 10% fetal bovine serum (FBS) in 5% CO_2_ in humidified incubator at 37°C. The cells were seeded in 12 well plates at a density of 3 x 10^4^ cells/well and grown for 24 hrs. The next day, the cells were treated with 20 μM DPA compounds at 50% confluency. The treated cells were incubated in 5% CO_2_ in humidified incubator at 37°C for 48 hrs.

### RNA Extraction, cDNA Synthesis and Real-time quantitative PCR

Total RNA for treated and untreated control was isolated using TRIzol reagent (Invitrogen). To quantify miRNA expression levels, equal amounts of cDNA were synthesized. The genomic DNA was eliminated using the gDNA wipeout buffer (Quiagen) at a reaction of 42°C for 2 min followed by snap cooling on ice. The RNA was polyadenylated with ATP by poly (A) polymerase at 37°C for 30 min and reverse transcribed using 0.5 μg of oligo (dT) adaptor primer by Revertaid reverse transcriptase enzyme (Thermo Fisher).

The real time qPCR was performed using SYBR Green PCR Mastermix (Kappa) on a LightCycler 480 Real-Time PCR System (Roche Applied Science). All qPCR reactions were conducted at 50°C for 2 min, 95°C for 10 min, and then 50 cycles of 95°C for 15 s and 60°C for 1 min. The specificity of the reaction was verified by melt curve analysis. Each miRNA were detected using miRNA specific forward primer (miR-142: 5′ CATAAAGTAGAAAGCACTACT 3′; miR-335: 5′ TCAAGAGCAATAACGAAAAATGT 3′; miR-504: 5′ AGACCCTGGTCTGCACTCTATC 3′) and 3′ universal reverse primer (5′ CTCAATCGTACATAGAAACAGGGATC 3′). Human small nuclear U6 small RNA was amplified as an internal control (FP: 5′ CGCAAGGATGACACGCAAATTC 3′) and all miRNA expression data were normalized to U6 small RNA expression. The analysis of miRNA expression was done using comparative delta delta Ct method [45].

## Results and Discussion

### Screening of 20 miRNAs for F-neo binding

**Fig 3:**
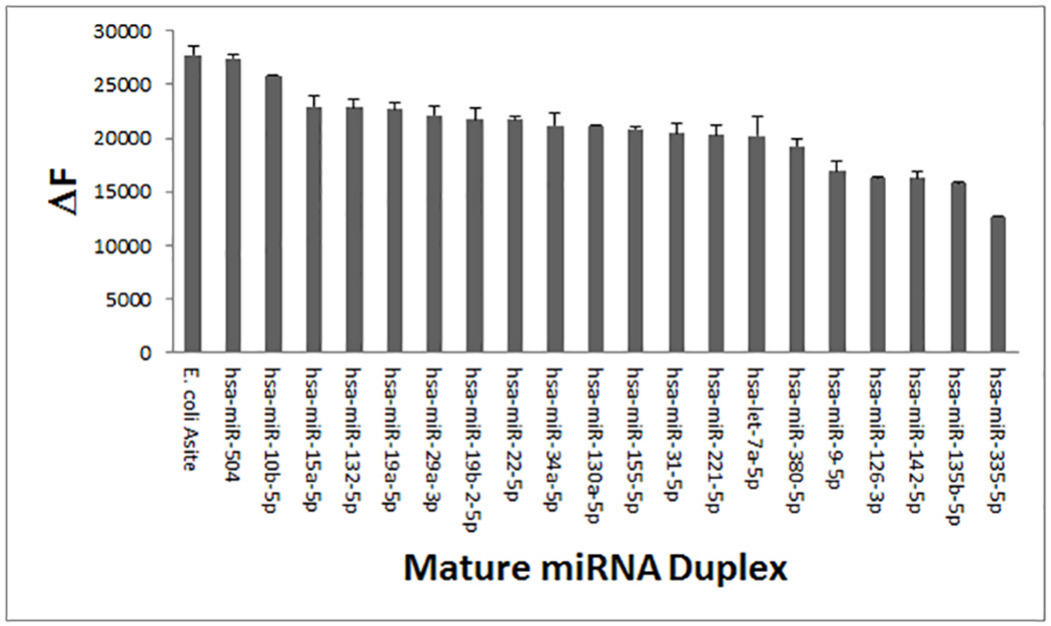
The screening of the 20 miRNA duplexes containing the mature miRNA sequence. The miRNAs were screened for the change in fluorescence with the addition of equal molar concentrations of the probe F-neo. The 27 base model of the *E*. *coli* A-site is included as a positive control. The miRNA with the highest change in fluorescence, hsa-miR 504, the tumour suppressing miRNA with the lowest change in fluorescence, has-miR 142, and the oncogene with the lowest change in fluorescence, has-miR 335, were chosen for assay development and compound screening.

A library of 20 hairpin miRNAs, each containing a mature sequence that is related to cancer as either an oncogene or a suppressor, were screened to determine the utility of F-neo as a general probe for miRNAs [46] (Fig 3). The initial screen of the miRNA library shows that F-neo binds to all of the mature miRNAs tested when added at a 1:1 ratio. The affinity of F-neo for all of the miRNAs tested indicates that F-neo could be used as a general fluorescent probe in a competitive binding assay for miRNAs. In order to demonstrate the application of F-neo as a general miRNA probe, we selected three miRNAs for further study: the miRNA with the greatest change in fluorescence, hsa-miR-504; the miRNA with the lowest change in fluorescence that is classified as an oncogene, hsa-miR 142; and the miRNA that has the lowest change in fluorescence that is classified as a tumor suppressor, hsa-miR 335. Additionally, we developed the assay for the pre-miRNA, pre hsa-miR 504, to show that the application can be extended to miRNAs at different stages of processing.

### The quenching of F-neo upon binding miRNA is a result of a shift in the pK_a_ of fluorescein

The fluorescein moiety of the F-neo probe has been shown to have different absorbance properties that are dependent on the protonated state of fluorescein[24]. The monoanion and dianion states are both potentially relevant at biological pH. The dianion strongly absorbs photons at 480 nm and gives a strong emission peak at 517 nm. The monoanion absorbs weakly at 480 nm and gives greatly reduced emission at 517 nm (Fig 2). The change in the absorbance properties of fluorescein as a function of protonation state has allowed the molecule to be used as a probe for changing pH and a probe of the electrostatic environment [47–49].

We have previously shown that F-neo has similar absorbance and fluorescence properties as fluorescein. These properties of F-neo make it ideally suited to monitor the binding of molecules to nucleic acids. Unlike other probes, which are quenched as a result of staking interactions with guanine, the quenching of F-neo results largely from the change in electrostatic environment [50–53]. The change in the electrostatic environment results in a shift in the pK_a_ of the fluorescein. Because the groove of nucleic acids has a more negative potential than that of the environment, the binding of F-neo to the nucleic acid has been shown to shift the pK_a_ to the non-fluorescent monoanion.

**Fig 4:**
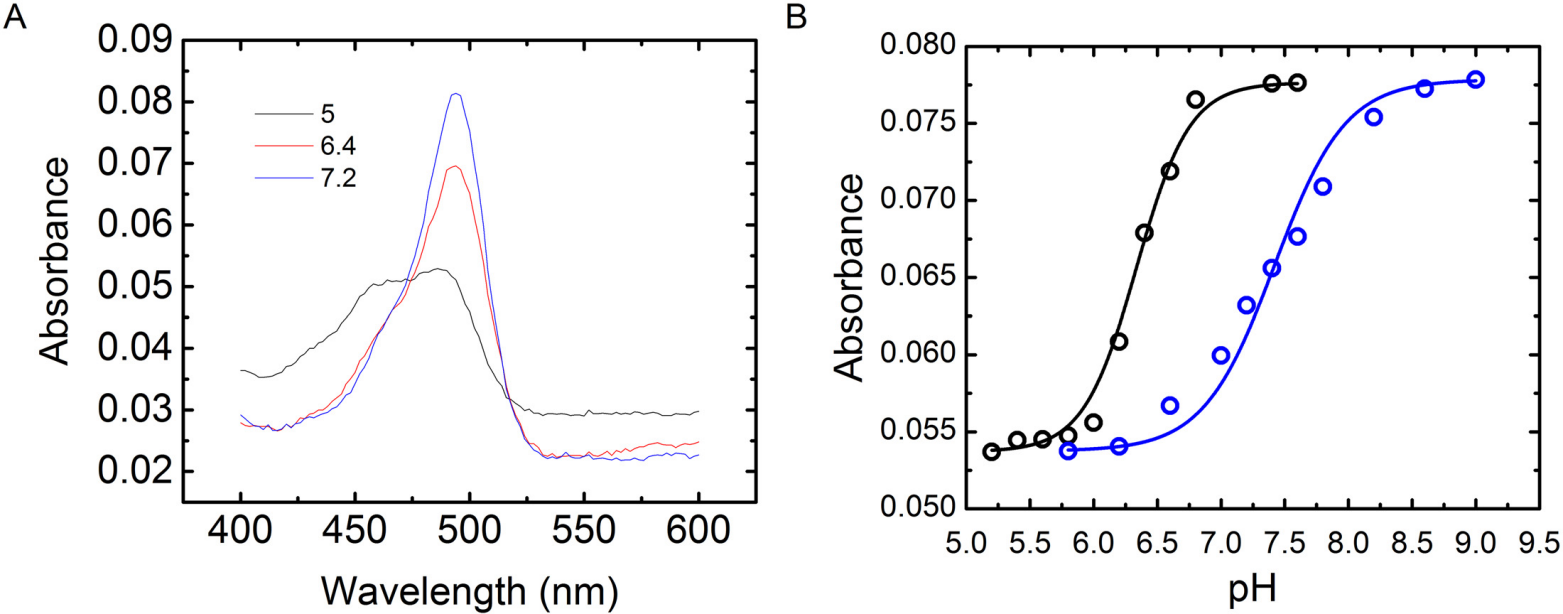
The shift in the pK_a_ of F-neo in the presence of mature duplex miRNA. A) The absorbance spectra of 1 μM F-neo in 10 mM NaPO_4_, 25 mM KCl, and 0.05 mM EDTA pH 7.2, 6.4, and 5.0. B) The absorbance at 490 nm of F-neo as a function of pH in the absence (black) and presence (blue) of the mature duplex hsa-miR 504.

The shift in the pK_a_ of F-neo has previously been shown to be the mechanism of quenching upon the binding of a 27 base model of the *E*. *coli* ribosomal A-site [54]. Here we demonstrate that a similar shift in the pK_a_ of F-neo occurs upon binding to miRNAs. In order to determine the mechanism of quenching of F-neo upon miRNA binding we first determined the pK_a_ under the binding conditions of miRNA by monitoring the absorbance spectrum as a function of pH for F-neo alone. A plot of absorbance at 490 nm versus pH results in a pK_a_ of 6.37 for the F-neo probe (Fig 4). This value corresponds to the pK_a_ of the non-conjugated fluorescein and is similar to that of the previously determined pK_a_ of F-neo [54]. Upon addition of an equivalent amount of the mature hsa-miR 504 duplex, the absorbance is significantly reduced at a pH of 6.5. The titration of pH results in a shift in the pK_a_ to 7.45 in the presence of hsa-miR 504 (Fig 4).

The electrostatic environment of the binding site of F-neo (φ) is directly proportional to the change in the pK_a_ of the probe in the bound and unbound state. The value of φ can be determined using Eq 3.

φ=−(2.303RTNAe) ΔpKa (3)

where R = 8.312 J mol^-1^ K^-1^, T = 298.2 K, N_A_ = 6.023 x 10^23^, and *e* = 1.602 x 10^−19^ C. Thus the binding site of F-neo has a φ of -2.46 kT/*e*. The large negative φ of the binding site is similar to that determined for the F-neo binding to the ribosomal A-site model [54], and indicates that F-neo binds in the groove of the mature hsa-miR 504 duplex in a similar manner.

### Determination of the binding affinity of F-neo for miRNA

**Fig 5:**
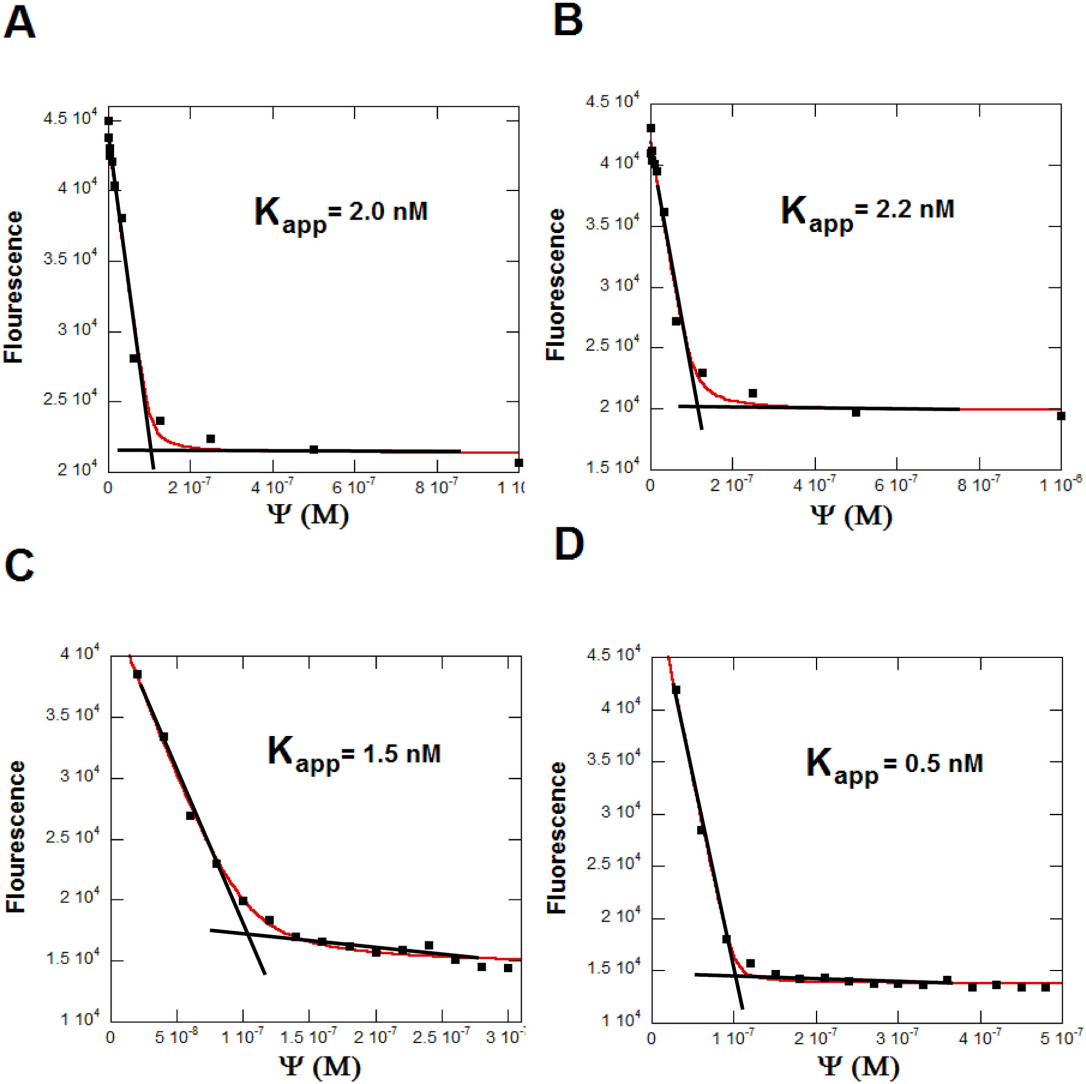
The determination of the apparent dissociation constant of F-neo from miRNA binding sites. Each miRNA was titrated into 100 nM F-neo. The titration was performed as the concentration of binding sites (Ψ). For (A) hsa-miR 142 and (B) hsa-miR 335, Ψ is equal to the number of moles of miRNA. (C) hsa-miR 504 has two binding sites on each molecule of miRNA, so Ψ is equal to two times the concentration of hsa-miR 504. (D) pre-hsa-miR 504 Ψ equals six times the concentration of miRNA.

An initial titration of F-neo from 0.5 nM to 1000 nM into miRNA was performed on all miRNA constructs. For the miRNAs hsa-miR 142 and hsa-miR 335 it was determined that saturation was reached at a 1 to 1 binding ratio. Due to the sharpness of the transition for hsa-miR 504 and pre-hsa-miR 504 the range of the titration was narrowed to the concentration range of 10 nM to 150 nM for the mature hsa-miR 504 and 5 nM to 80 nM for the pre-hsa-miR 504 to better isolate points within the transition and the binding curve. The binding curve of the mature hsa-miR 504 reached saturation at two molecules of F-neo to one molecule miRNA. As expected, the longer pre-hsa-miR 504 contains multiple binding sites for F-neo, reaching saturation at six probe molecule for every pre-hsa-miR 504 (Fig 5).

**Fig 6:**
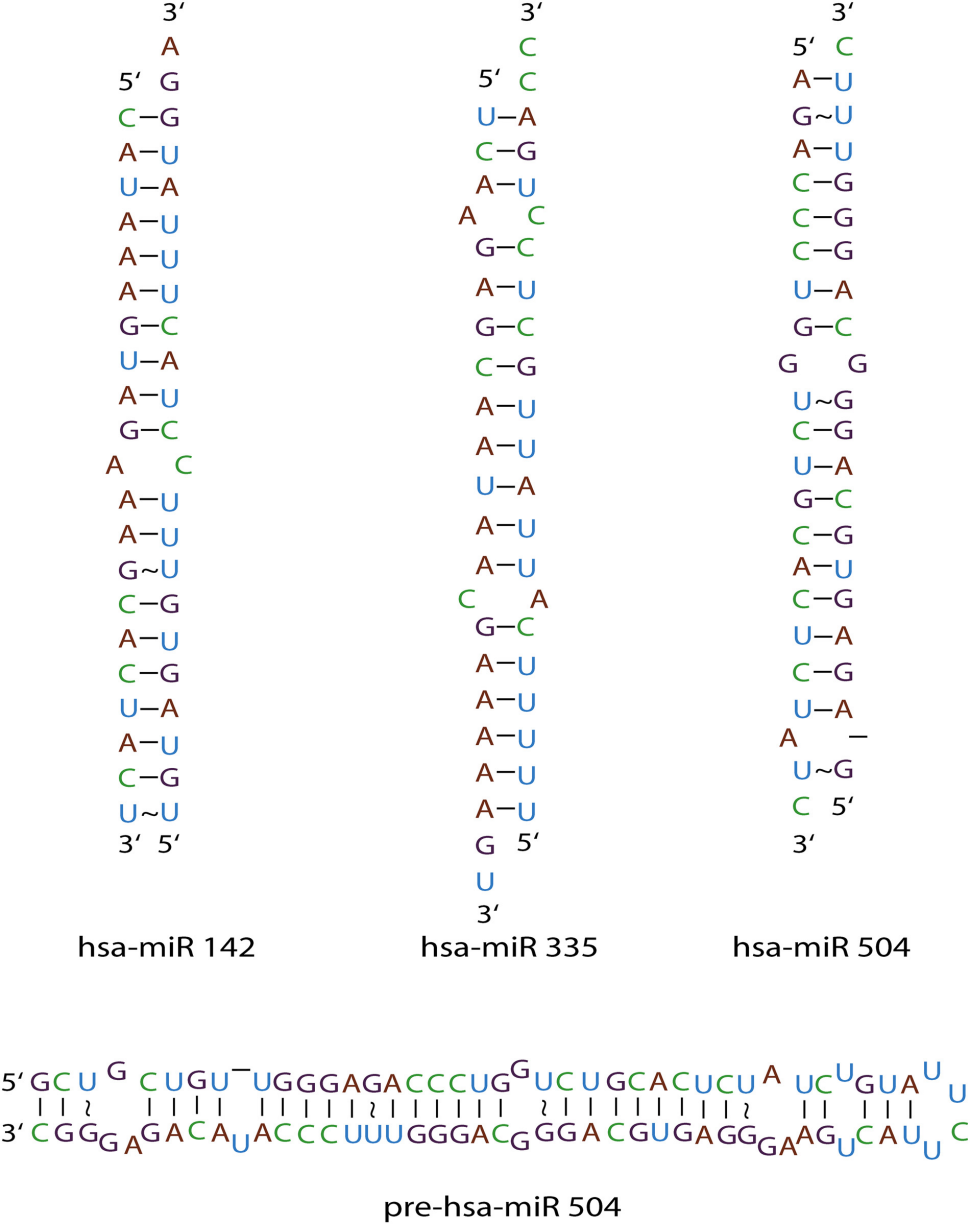
The secondary structure of mature duplex miRNAs and Pre-miRNA screened with the F-neo displacement assay for binding with PA library. The secondary structure predictions were taken miRbase [56–60]. Mature duplex miRNAs were constructed by the removal of bases from the pre-miRNA predictions not included in the mature sequence.

The structure of the miRNAs consists largely of canonical base pairs, but do contain regions of non-Watson Crick base pairing. The number of binding sites correlates well with the number of non-canonical regions predicted by the secondary structure of the miRNAs. The bulges in the miRNAs are the likely binding sites for F-neo, neomycin, and the neomycin-amino acid conjugates. The predicted secondary structure of the mature hsa-miR 504 has two bulges which correlates well with the number of binding sites observed (Fig 6). The pre-hsa-miR 504 has six bulge regions in the secondary structure, which again correlates well with the number of binding sites, and hsa-miR 142 has only a single bulge in the secondary structure and only a single binding site is observed. The correlation is not perfect in our small sample however; two bulges are observed in hsa-miR 335, but only one binding site is observed.

The mature and pre-hsa-miR 504 both have multiple F-neo binding sites. In order to estimate the binding affinity, we treated all binding sites as equivalent and established an apparent K_D_ for each binding. This assumption is based on the fact that the titration with F-neo leads to a single transition. The single transition indicates that the affinity of F-neo for each binding site is similar Fig 5). Additionally, the resulting apparent K_D_ was similar for both hsa-miR 142 and hsa-miR 335. Therefore, the binding curve was determined as the concentrations of binding sites (Ψ) versus the change in fluorescence. This treatment gives two binding sites for every molecule of the mature hsa-miR 504 and six binding sites for every molecule of the pre-miR 504, and apparent K_D_’s of 1.5 nM for the mature sequence and 0.5 nM for the pre-hsa-miR 504 were extrapolated. Since both hsa-miR 142 and hsa-miR 335 have a single binding site, Ψ is equal to the concentration of miRNA, and the K_D_ was determined by fitting of the F-neo titration data to Eq 1 (Fig 5). The fit of the equation resulted in a K_D_ of 2 nM for hsa-miR 142, and 2.2 nM for hsa-miR 335 (Table 1).

**Table 1:** Summary of miRNA binding results.

	hsa-miR 142	hsa-miR 335	hsa-miR 504	Pre-hsa-miR 504
F-neo K_D_ (nM)	2.0	2.2	1.5	0.5
Neomycin IC_50_ (nM)	95.7 ± 5.6	117.4 ± 8.1	70.3 ± 1.6	56.2 ± 2.3

There are apparent structural and/or chemical differences in the binding sites of the miRNAs. The quenching of the fluorescein of the F-neo probe is largely a function of the change of the electrostatic environment of the probe [47,54]. The change in fluorescence of hsa-miR 142 and hsa-miR 335 is only ~70% of that of hsa-miR 504. The fluorescence intensity difference observed upon probe binding indicates that binding site is physically and/or chemically different in the miRNAs. The difference in the miRNA binding sites offers the potential to target a specific miRNA.

### Determination of the binding affinity of neomycin by F-neo displacement

The IC_50_ of neomycin was calculated for the displacement of F-neo from the mature and pre-hsa-miR 504, mature hsa-miR 142, and mature hsa-miR 335 by neomycin. Neomycin was then titrated into the miRNA:F-neo complex based on a one binding site per F-neo molecule model. Therefore, hsa-miR 142 and hsa-miR 335 were both at a 1:1 molar ratio to F-neo. The mature hsa-miR 504 was combined with F-neo at a 1:2 ratio, and the pre-hsa-miR 504 was set at a 1:6 ratio with F-neo.

As with the determination of the K_D_ of F-neo, all neomycin binding sites were treated as equivalent binding sites. The treatment as such defines the IC_50_ as the concentration of neomycin necessary to displace 50% of the F-neo from the binding sites. The fitting of the sigmoidal binding curve results in an IC_50_ of 95.7 nM for hsa-miR 142 binding sites, an IC_50_ of 117.4 nM for hsa-miR 335 binding sites, an IC_50_ of 70.3 nM for the mature hsa-miR 504 binding sites, and an IC_50_ of 56.2 nM for pre-hsa-miR 504 binding sites (Table 1). The affinity of F-neo is similar for miRNAs hsa-miR 142, hsa-miR 504, and pre-hsa-miR 504, and approximately 50% higher for hsa-miR 335. Additionally, the relative affinity of neomycin is similar for all miRNAs indicating that neomycin may be a general miRNA binding molecule, and this generality may extend to other similar aminoglycosides.

### Development of a high throughput displacement assay of F-neo from miRNA by neomycin

**Fig 7:**
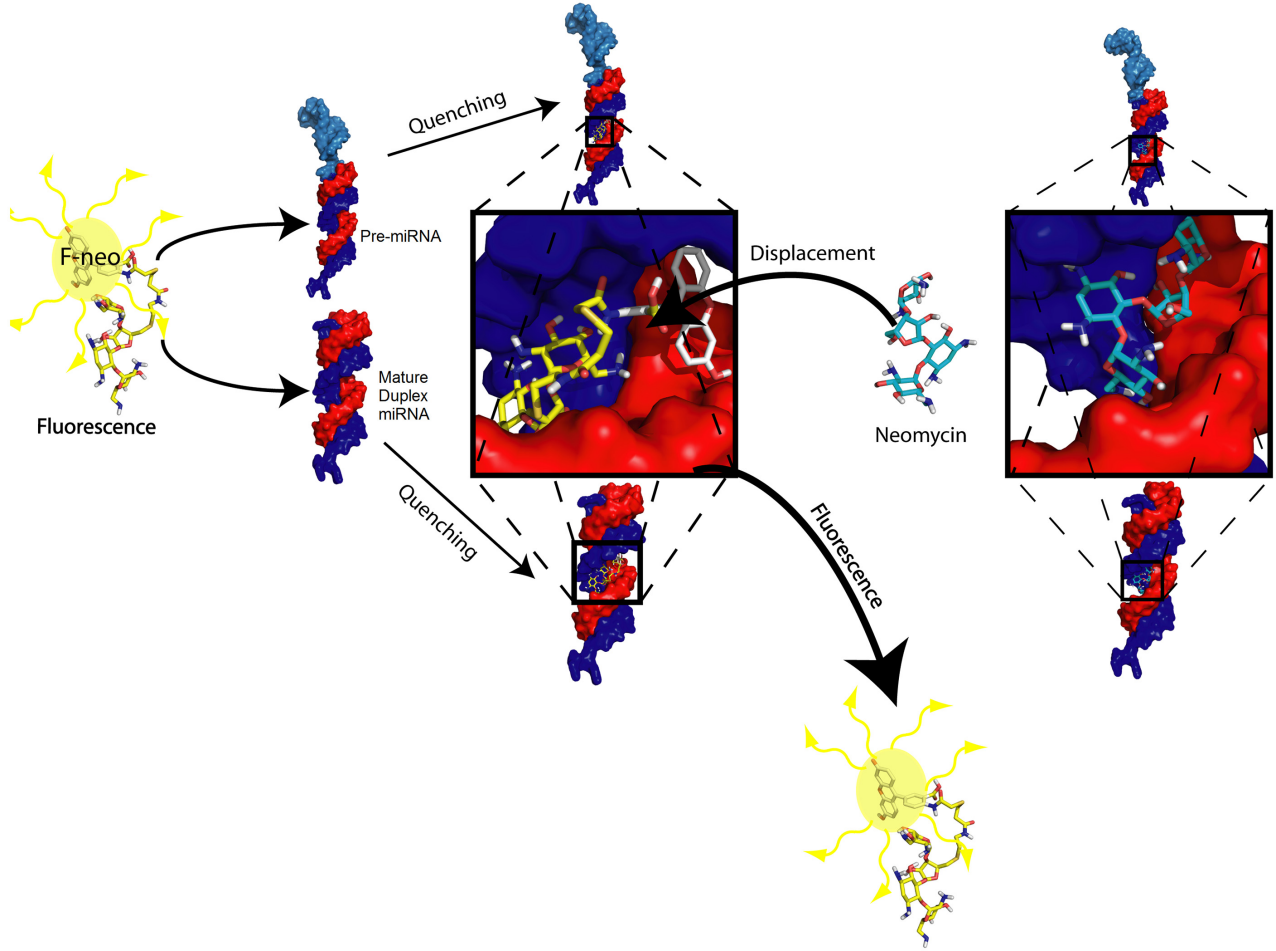
Schematic of F-neo displacement assay for miRNA binding compounds. The fluorescence of F-neo (yellow, stick; fluorescence indicated when present) is quenched when bound in the groove of either the mature duplex or pre-miRNA. The addition of a miRNA binding compound, neomycin (cyan, stick), displaces the F-neo. The displacement of F-neo results in an increase in fluorescence. The mature miRNA is shown as a red (5’ strand) and blue (3’ strand) surface, and the addition of the loop region shown as a sky blue surface represents the pre-miRNA. The location of the binding site on the miRNA is highlighted with a black box was identified by docking experiments using Autodock Vina [61].

A high throughput screen for compounds that bind miRNA was developed in a 96-well format based on the displacement of F-neo by neomycin (Fig 7). The assay was developed for the mature miRNAs hsa-miR142, hsa-miR 335, and has-miR 504, as well as the pre-hsa-miR 504. The neomycin binding curves indicated that a concentration of three to five times the concentration of F-neo would give the optimum signal window for all miRNAs. The optimal conditions were determined by the calculation of the Z’-factor using 48 wells of the positive control (F-neo miRNA + neomycin) and 48 wells of the negative control (F-neo + miRNA) using Eq 2. The quality of the assay was determined using the accepted scale of the Z’-factor: >0.5 is an excellent assay, <0.5>0 is a marginal assay, and <0 is unacceptable [55].

The mature and pre-hsa-miR 504 miRNAs assays were both optimized using three times the concentration of neomycin as compared with that of F-neo. The Z’ factor for mature hsa-miR 504 was 0.60 at 300 nM neomycin to 100 nM F-neo and 50 nM miRNA. The Z’ factor for the pre-hsa-miR 504 was 0.64 at 300 nM neomycin to 100 nM F-neo and 16.7 nM miRNA.

The signal window for the hsa-miR 142 and hsa-miR 355 was not sufficient to give a Z’ factor above 0.5 using 300 nM neomycin for the displacement of 100 nM F-neo. The assay for these miRNAs was optimized to 500 nM neomycin in order to increase the signal upon displacement. The optimal conditions for both hsa-miR 142 and hsa-miR 335 were determined by the calculation of the Z’-factor using 48 wells of the positive control (100 nM F-neo + 100 nM miRNA + 500 nM neomycin) and 48 wells of the negative control (100 nM F-neo + 100 nM miRNA) using Eq 2. The Z’ factor for miRNA 142 was 0.50 at 500 nM neomycin to 100 nM F-neo and 100 nM miRNA. The Z’ factor for miRNA 335 was 0.51 at 500 nM neomycin to 100 nM F-neo and 100 nM miRNA.

The Z’-factor established for the optimized assays for all miRNAs indicate that the assay are suitable for screening of compounds. The assay is based on the displacement of F-neo by a competitive binding compound at a compound concentration of 300 nM for hsa-miR 504 and pre-hsa-miR 504 and a compound concentration of 500 nM for hsa-miR 142 and hsa-miR 335.

### Screening of the PA library for miRNA binding

In order to improve specificity and target specific miRNAs, the miRNA binder neomycin was conjugated to different amino acids to create the peptide-aminoglycoside (PA) library. The conjugation to amino acids can be done rapidly to produce compounds with a wide range of physical and chemical properties, including various charges, hydrophobicity and size. A library of 215 amino acid conjugated neomycin compounds containing mono- and di- amino acid conjugates to neomycin was screened for binding by the F-neo displacement assay to the three mature miRNAs: hsa-miR 504, hsa-miR 335, and hsa-miR 142, and also the pre-hsa-miR 504. All compounds in the library were screened in duplicate with each of the miRNAs, and the average binding of each compound was normalized to the binding of neomycin using Eq 4.

$$\%\;binding\;=\;\left(\Delta F_{drug}/\Delta F_{neomycin}\right)\;x\;100\%$$ (4)

Where ΔF_drug_ is the difference in the fluorescence between the F-neo:miRNA complex only and the F-neo:miRNA complex in the presence of the indicated compound. ΔF_neomycin_ is the difference in the fluorescence between the F-neo:miRNA complex only and the F-neo:miRNA complex in the presence of neomycin.

The average binding of all compounds to each miRNA gives an overall indication of the library’s affinity for each miRNA. For a number of PAs, the affinity of the compound resulted in a lower affinity than that of neomycin alone. The average highest affinity of the library was 72% for the mature hsa-miR 504, followed closely by the pre-hsa-miR 504 sequence at 67% for all compounds within the library. The overall average of 53% was significantly lower for hsa-miR 142, which was similar to that of hsa-miR 335 at 51% (Table 2; full results with error given in S1 Table). The average binding for all compounds is 70% for pre and mature hsa-miR 504, compared with ~50% for hsa-miR 142 and hsa-miR 335, demonstrate that the pre and mature duplex hsa-miR 504 binding sites differ from those of the mature duplex hsa-miR 142 and hsa-miR 335.

**Table 2:** Average binding of all Neomycin conjugates to miRNAs.

	hsa-miR 142	hsa-miR 335	hsa-miR 504	Pre-hsa-miR 504
Average Percent Displacement for all NeoXX* compounds	53%	52%	72%	67%

*X indicates the variable amino acids A, R, N, D, H, L, F, P, S, T, Y, V, C, W. The amino acid K is included for in X for NeoX, NeoXS, NeoXT, NeoXY, and NeoXV. For data on percent binding and σ for all compounds, please refer to S1–S9 Tables.

The screen identified several compounds that bind to miRNA better than the parent neomycin. These high affinity compounds only bind with higher affinity to the constructs of hsa-miR 504. Surprisingly, six compounds, NeoAS, NeoRA, NeoRH, NeoNS, NeoFS, NeoVP, bind with higher affinity than neomycin to the mature duplex hsa-miR 504, and only three compounds, NeoNY, NeoRS, NeoRV, bind with higher affinity to pre-hsa-miR 504. These results indicate that the PA compounds demonstrate slightly different selectivity for the pre-miRNA sequences as compared with the processed mature duplexes.

### Verification of the single point screen by IC_50_ determination

**Fig 8:**
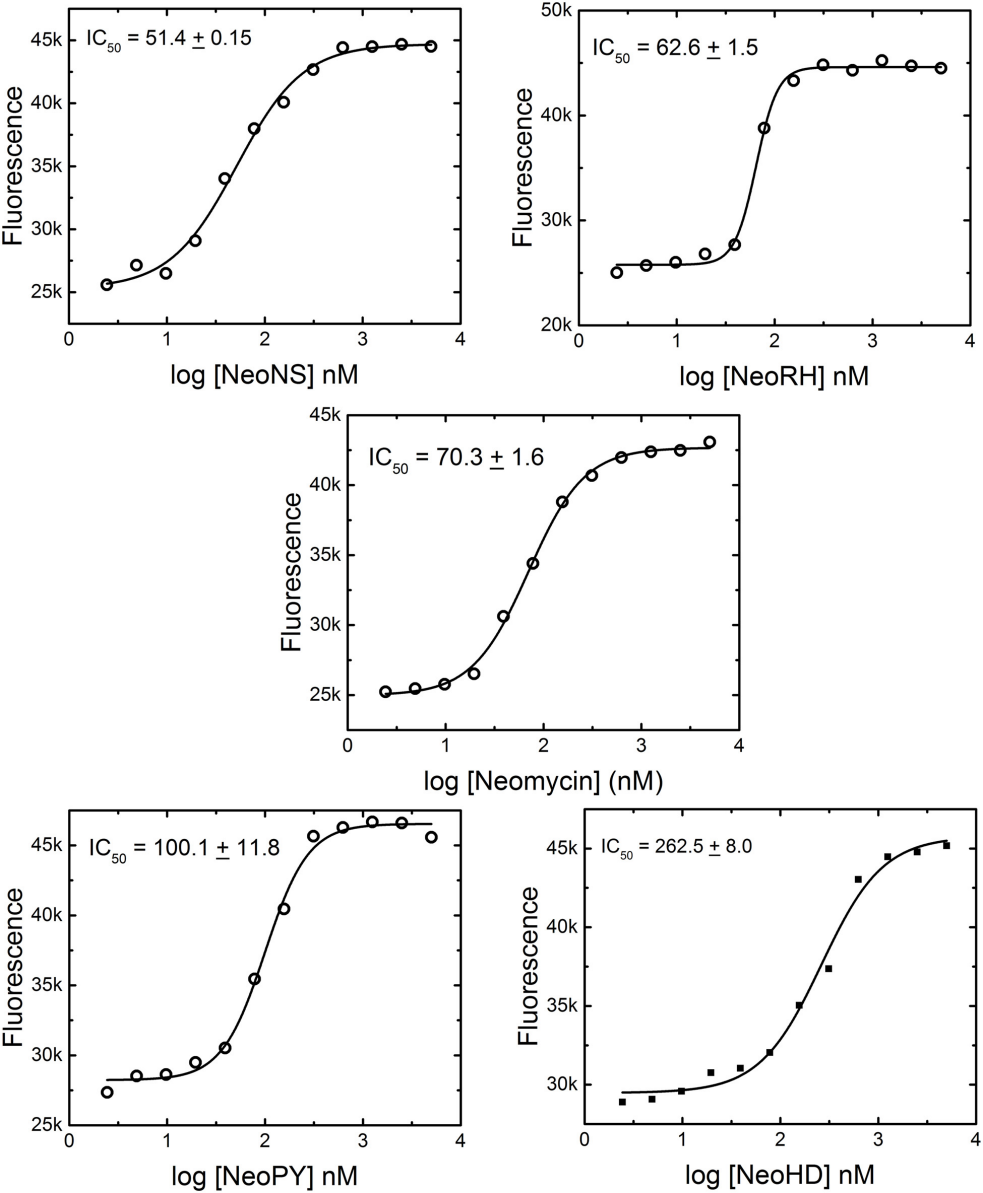
The IC_50_ determination of compounds for mature duplex hsa-miR 504. The IC_50_ for each compound was determined from a sigmoidal fit of a titration of the designated compound into 100 nM F-neo, 50 nM mature duplex hsa-miR 504 in 10 mM NaPO_4_, 25 mM KCl, 0.05 mM EDTA at pH 6.5. All measurements were taken in a 96-well black Greiner plate using a Tecan Infinite M-1000 plate reader with an excitation of 485 nm and an emission of 525 nm.

In order to validate the single point assay’s ability to accurately determine the relative affinity of compounds for their target miRNAs, the IC_50_ values for a subset of compounds were determined for the displacement of the F-neo probe by compounds from the PA library using the mature duplex hsa-miR504 as a substrate. The IC_50_ was determined for two compounds that have a higher binding percentage as compared with neomycin, NeoNs and NeoRH, and two compounds that have a lower binding percentage as compared with neomycin, NeoPY and NeoHD (Fig 8). The IC_50_ for each compound was compared to the binding percentage. As shown in Table 3, both assays rank the compounds’ affinity in the same order, with NeoNS and NeoRH having lower IC_50_s than neomycin and NeoPY and NeoHD having greater IC_50_s. These results indicate that the single point screen is capable of accurately predicting the relative binding affinity of a compound library for a specific miRNA.

**Table 3:** The ranking of binding affinity for select PA compounds for hsa-miR 504.

Compound	% Binding	IC_50_ (nM)
NeoNS	127 ± 14	51.4 ± 0.15
NeoRH	121 ± 12	62.6 ± 1.5
Neomycin	100	70.3 ± 1.6
NeoPY	50 ± 8	100.1 ± 11.8
NeoHD	9 ± 2	262.5 ± 8.0

### Preference of miRNAs for specific amino acids

In order to establish a preference for an amino acid at different positions of the PA library, we determined a relative binding affinity for a particular target miRNA as a function of the amino acid at each position. While the average effect of the conjugation reduces the affinity of neomycin for the miRNAs, some amino acids have a greater impact on the affinity than others. Additionally, the position of the amino acid also influences the compounds’ affinities. The evaluation of the preference of the amino acids at each position for may provide information about the binding site of each miRNA and indicate which conjugates are best suited for further modification to increase affinity and specificity.

In order to normalize the compound library for each miRNA, we determined the average standard deviation of binding of each compound from the mean (σ). We then determined the number of standard deviations from the mean for each compound for each miRNA. The results are summarized in Table 4 and full results of these calculations are given in S6–S9 Tables. A positive σ indicates that an amino acid residue at that position has a higher affinity than the average of the PA library for the indicated miRNA and a negative σ indicates a reduction in the affinity of conjugate compared to the other conjugates for the indicated miRNA.

**Table 4:** Compound classes with large deviations from the mean binding of the compound library.

	hsa-miR 142	hsa-miR 335	hsa-miR 504	Pre-hsa-miR 504
σ for all NeoXX compounds	21%	20%	23%	20%
Compounds σ averaged over all variations at position 1 and no amino acids at position 2.^a^				
NeoX*	-0.43	0.80	0.26	-0.09
Compounds σ averaged over all variations at position 2 with constant amino acids at position 1.^a^				
NeoRX	0.64	0.75	0.63	1.06
NeoDX	-0.92	-0.94	-1.05	-1.14
NeoWX	-0.43	-0.54	-0.38	-0.20
Compounds σ averaged over all variations at position 1 with constant amino acids at position 2.^a^				
NeoXD	-1.22	-1.38	-1.52	-0.70
NeoXW	-0.67	-0.59	-0.36	-0.12
NeoXS	1.21	0.21	1.20	0.90
NeoXT	0.95	0.58	0.17	0.45
NeoXY	0.61	0.78	0.26	0.39
NeoXβA	-0.57	0.55	-0.42	-0.41
NeoXH	-0.55	-0.25	0.80	-0.04
NeoXR	0.13	0.74	0.36	-0.34
NeoXL	-0.27	-1.05	0.00	-0.71
NeoXF	0.32	-0.88	-0.17	-0.41
NeoXC	0.11	0.40	-0.66	-0.46
NeoXV	0.17	0.30	0.28	0.78

^a^The average binding and standard deviation from the mean (σ) was taken for all compounds in the library. The σ for the binding was determined for each compound. The average σ for all compounds with the indicated amino acid was determined for all compounds variations at position X.

*X indicates the variable amino acids A, R, N, D, H, L, F, P, S, T, Y, V, C, W. The amino acid K is included for in X for NeoX, NeoXS, NeoXT, NeoXY, and NeoXV. For data on percent binding and σ for all compounds, please refer to S1–S9 Tables.

By comparing the σ of each compound for each miRNA we can normalize our results to account for the general effects of conjugating the amino acids to neomycin, such as increasing the size of neomycin, has on each miRNA. For instance, the average of the percent displacement for all compounds with histidine as the first amino acid conjugated to neomycin (NeoHX) is 77% for mature duplex hsa-miR 504 and 57% for mature duplex hsa-miR 142, which would indicate a more significant impact for histidine conjugates on the binding of hsa-miR 504 as compared with hsa-miR 142. However, the change in the deviation from the mean is .24σ for mature duplex hsa-miR 504 and 0.15σ for mature duplex hsa-miR 142. The small change in σ indicates that the identity of the amino acid at position 1 has a similar impact on affinity for both miRNAs. Thus, an increase in a conjugate’s σ for miRNA and a decrease in same conjugate’s σ for another miRNA would indicate an increase in specificity of the compound for a specific miRNA as a result of the conjugation of specific amino acid(s). Therefore, the change in the value σ for each compound was considered when analyzing the significance of each amino acid at each position in the neomycin conjugates.

The standard deviation from the mean (σ) was ~20% for all miRNAs, indicating that the presence of an amino acid disrupted the binding for hsa-miR 142 and hsa-miR 335 more than that of hsa-miR 504, but on average the identity of the amino acid had little difference in the disruption after the initial cost. Additionally, differences in the response were uniform for residues in the first amino acid position, but differences in the influence between the miRNAs began to appear with the presence of the second amino acid (S6–S9 Tables). With as few as two amino acids conjugated to neomycin, a difference was observed on the influence of the identity of the amino acids, in that individual compounds were observed that increased the binding for specific miRNAs.

An arginine residue at position 1, NeoRX (NeoRX, where X is any or no amino acid in position 2), appears to be important for the affinity for all miRNAs. NeoRX demonstrated the highest affinity on average for all miRNAs as compared to other amino acids at position 1, with at least one of the NeoRX conjugates retaining or increasing the binding of this conjugate over neomycin. This suggests the presence of the positively charged arginine may partially compensate for the steric interactions associated with the increased size of neomycin by the amino acid side chains. The increase above the mean for the single substituted neomycin-amino acid conjugate, NeoR, for hsa-miR 142 (2.04σ) and hsa-miR 335 (2.18σ) shows that it has a positive influence on binding compared to other residues. Similarly, NeoR appears to enhance the binding of mature hsa-miR 504 (1.84σ) and pre-hsa-miR 504 (1.76σ). The derivatives of NeoRX have an average of 0.64σ– 1.06σ indicating that arginine is the most beneficial residue at position 1.

The negatively charged aspartate conjugates NeoDX resulted in a significant drop in affinity for all miRNAs, reducing the affinity for hsa-miR 142 by -0.92σ, hsa-miR 335 by -0.94σ, pre-hsa-504 by -1.14σ, and hsa-miR 504 by -1.04σ compared to the average binding for all conjugates. The NeoXD conjugates were equally destabilizing, reducing the affinity hsa-miR 142 by -1.22σ, hsa-miR 335 by -1.38σ, pre-hsa-504 by -0.70σ, and hsa-miR 504 by -1.52σ. While less destabilizing than aspartate, the tryptophan conjugates, NeoWX and NeoXW, both resulted in average negative binding impact on all miRNAs. These results indicate that negative residues and large bulky groups may be harmful in the binding of many miRNAs.

The impact of amino acids at position 1 was consistent for all miRNAs, but the impact at position 2 is more variable. The influence on binding by a second amino acid may indicate that the influence on binding by the addition of amino acids can be obtained at sites that are at a distance away from the binding site of the neomycin. For hsa-miR 142, NeoXS, NeoXT, and NeoXY were 0.6σ or higher in binding. NeoXβA, NeoXH, and NeoXW had negative effects with -0.55σ or lower in binding.

Similar to hsa-miR 142, NeoXT and NeoXY had positive effects on affinity for hsa-miR 335 and NeoXW had negative effects on affinity for hsa-miR 335. However in general, hsa-miR 335 had the greatest positive effect from not having an amino acid at position 2, and by NeoXR and NeoXβA, which had a negative effect on affinity for hsa-miR 142. Additionally, compounds NeoXL and NeoXF had negative impacts on binding for hsa-miR 335. Additionally, a serine at position 2 (NeoXS) of the neomycin conjugates maintains or increases the binding of many of these conjugates for all miRNAs with the exception of hsa-miR 335. The exception of hsa-miR 335 to the trend of NeoXS conjugates may indicate a general difference in the binding site for these compounds for this miRNA compared to the other miRNAs.

The impact of the amino acid at position 2 was less pronounced for mature hsa-miR 504 than for the other mature miRNAs. Only four amino acids had an average σ above 0.5 when present at position 2 and the absence of an amino acid at position 2 was close to the average binding of all compounds. NeoXS has the most positive influence for binding hsa-miR 504, and as compared to all other miRNAs presented here, NeoXH had a positive impact on binding. In addition to the common negative impact of NeoXD, only NeoXC had a negative impact over -0.5σ at -0.66σ.

The pre-hsa-miR 504 contains the mature hsa-miR 504 sequence, so it is not surprising that these miRNAs demonstrate the most similar compound affinity. NeoXD and NeoXC class of compounds both have negative impacts on binding and NeoXS has a similarly positive impact on binding for the pre-miRNA as the mature hsa-miR 504. However, NeoXH class has an average σ that is close to zero for pre-hsa-miR 504. The NeoXV class is significantly higher for pre-hsa-miR 504 than for the other miRNAs tested.

**Fig 9:**
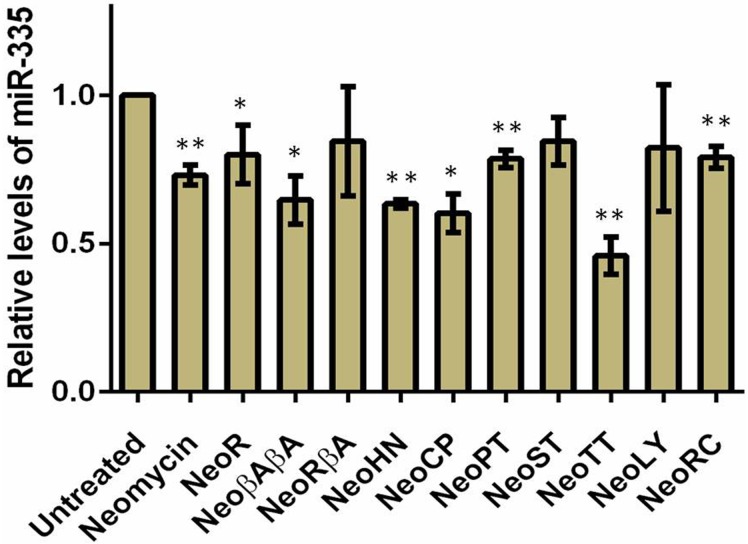
Relative expression of miR-142 levels after treatment of 20 uM DPA compounds for 48 hrs. The miRNA levels are normalized to U6 snRNA. Data are plotted as mean ± SEM *p < 0.05; **p < 0.01, unpaired two-tailed *t* test, n = 3.

**Fig 10:**
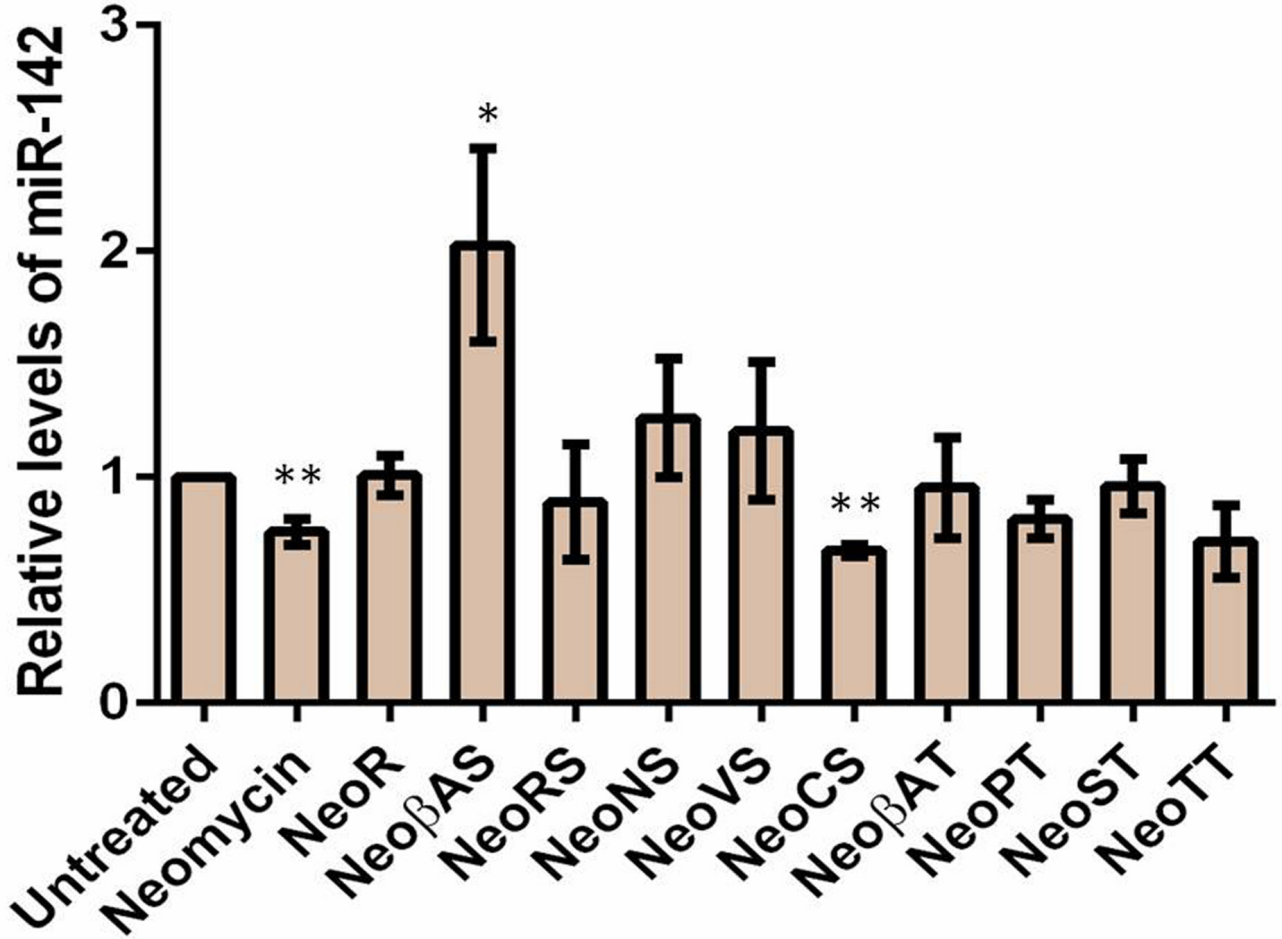
Relative expression of miR-335 levels after treatment of 20 uM DPA compounds for 48 hrs. The miRNA levels are normalized to U6 snRNA. Data are plotted as mean ± SEM *p < 0.05; **p < 0.01, unpaired two-tailed *t* test, n = 3.

**Fig 11:**
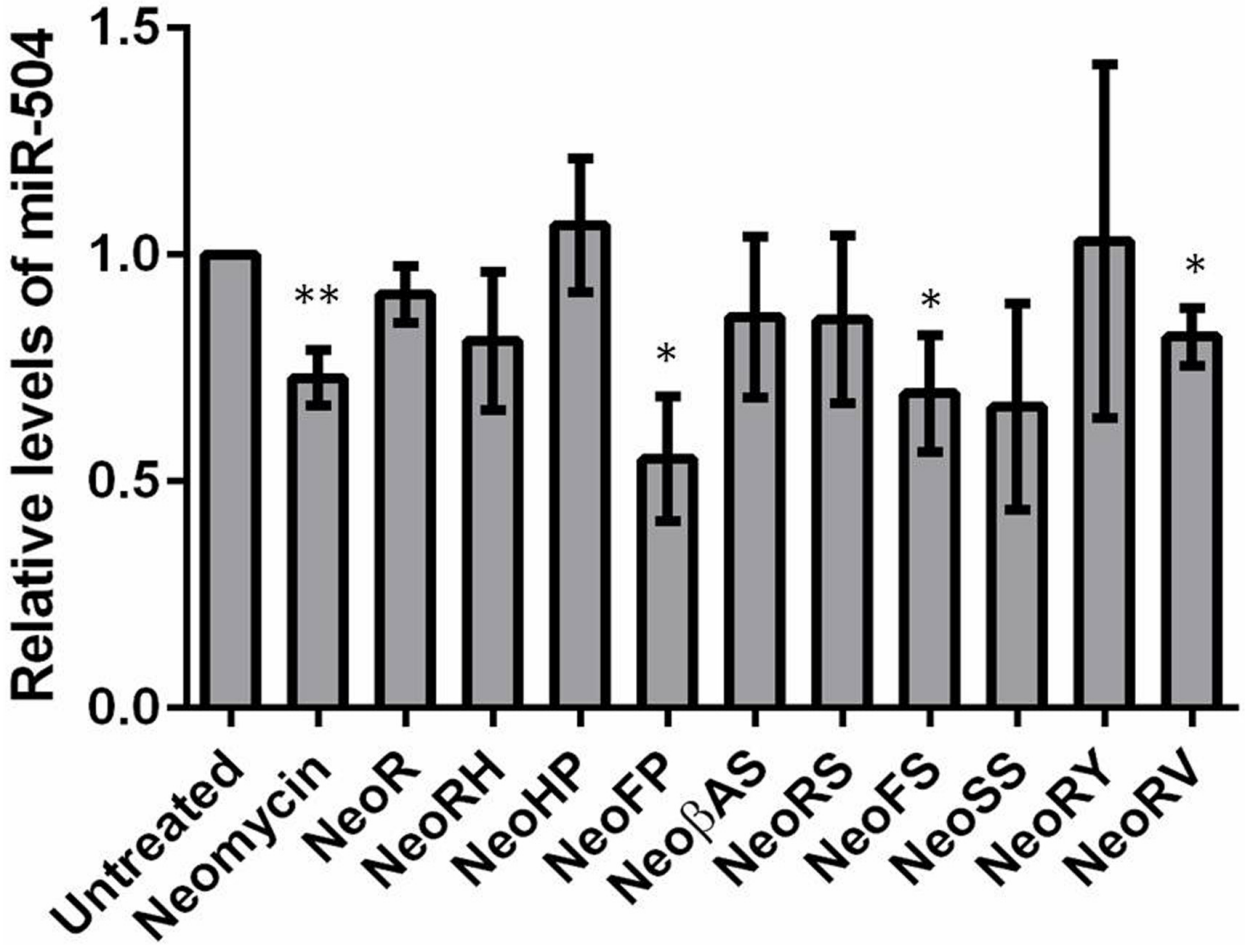
Relative expression of miR-504 levels after treatment of 20 uM DPA compounds for 48 hrs. The miRNA levels are normalized to U6 snRNA. Data are plotted as mean ± SEM *p < 0.05; **p < 0.01, unpaired two-tailed *t* test, n = 3.

Finally, we measured the expression profiles of miR-142, miR-335 and miR-504 by employing qPCR method following treatment with 20 μM of each DPA compounds in MCF-7 cell line for 48hrs. MiR-142, miR-335 and miR-504 were downregulated by neomycin treatment. MiR-142 showed a significant decrement upon treatment of neomycin derivative, DPA1240 whereas a ~2 fold upregulation after DPA1228 treatment (Fig 9). This can be reasoned by the fact that DPA1228 binding to pre-miR-142 might cause a structural perturbation such that it enhances Dicer activity leading to more production of mature miRNA levels. In case of miR-335, neomycin derivatives DPA1116, DPA1148, DPA1226, DPA1249, DPA 1251, and DPA1285 showed down regulation of miR-335 levels significantly (Fig 10). DPA1220, DPA1234 and DPA1271 potently reduced miR-504 levels thus validating these compounds as potential pre-miRNA binders (Fig 11).

## Conclusion

The discovery of miRNAs has presented a new therapeutic target. Here we have shown the development of a new approach for specific miRNAs targeting by small molecules. By using the F-neo displacement assay to screen a large compound library, we have shown that this assay can be used in the identification of compounds that bind with high affinity to different miRNAs at different stages of processing. From these results, we expect that this assay can be generalized to most miRNAs.

Here, we have screened a PA library of compounds for binding affinity of four different miRNA constructs, mature duplex hsa-504, hsa-miR 335, hsa-miR 142, and pre-hsa-miR 504. From the screening we identified eight compounds that bind with at least ten percent higher of affinity to the mature duplex hsa-miR 504 than the parent neomycin compound, and two compounds that bind with at least ten percent higher of affinity to the pre-hsa-miR 504 than the parent neomycin compound. Binding of PA compounds is also associated with downregulation of mature miRNAs in MCF-7 cells, presumably due to pre-miRNA binding and subsequent interference with pre-miRNA processing complexes.

While differences in the affinity of the probe for different miRNAs may make it difficult to predict the relative affinities of the compounds for different miRNAs, the screen does allow us to identify the highest affinity compounds for a specific miRNA, and allows us to correlate which modifications, such as the conjugation of amino acids to neomycin in this case, have the greatest impact. By comparing the impact of the conjugations on different miRNAs it may then be possible to identify which modification is most appropriate for a specific miRNA.

The difference in the miRNA binding sites appears to be both chemical and physical. The addition of amino acids to neomycin has the general effect of disrupting the binding of some miRNAs more than others, indicating that this disruption could be steric by nature and influenced by the size and/or shape of the binding pocket. However, the difference observed in the response to the addition of different amino acids shows a potential difference in the chemical nature of the binding site.

The approach described here using a library of aminoglycoside-amino acid conjugates and the high throughput F-neo displacement assay could lead to the identification of compounds that bind with high affinity and selectivity for a specific miRNA. The largest difference in the affinity was observed by only adding the amino acid to the neomycin. This specificity may be altered by the use of different aminoglycoside conjugates. Finally, greater specific influence on the binding of miRNAs was observed by the addition of a second amino acid. More specificity could potentially by obtained by extending the peptide chain to the aminoglycoside and such libraries are currently under investigation. Combining such an approach of altering the aminoglycoside and the peptide chain is expected to lead to compounds with desired affinity and specificity for any miRNA target, in addition to improved cell uptake.

## Supporting Information

S1 Table DPA numbered compounds with percent binding of neomycin. (DOCX)

S2 Table hsa-miR 142 Percent Binding of Neomycin. (DOCX)

S3 Table hsa-miR 335 Percent Binding of Neomycin. (DOCX)

S4 Table hsa-miR 504 Percent Binding of Neomycin. (DOCX)

S5 Table Pre-hsa-miR 504 Percent Binding of Neomycin. (DOCX)

S6 Table hsa-miR 142 Standard deviation from the mean of all compounds (σ). (DOCX)

S7 Table hsa-miR 335 Standard deviation from the mean of all compounds (σ). (DOCX)

S8 Table hsa-miR 504 Standard deviation from the mean of all compounds (σ). (DOCX)

S9 Table Pre-hsa-miR 504 Standard deviation from the mean of all compounds (σ). (DOCX)

## References

[1] Ghildiyal M, Zamore PD. Small silencing RNAs: an expanding universe. Nat Rev Genet 2009 2;10(2):94–108. 10.1038/nrg2504 19148191 PMC2724769

[2] Bartel DP. MicroRNAs: genomics, biogenesis, mechanism, and function. Cell 2004 1 23;116(2):281–297. 14744438 10.1016/s0092-8674(04)00045-5

[3] Lee RC, Feinbaum RL, Ambros V. The C. elegans heterochronic gene lin-4 encodes small RNAs with antisense complementarity to lin-14. Cell 1993 12 3;75(5):843–854. 8252621 10.1016/0092-8674(93)90529-y

[4] Wightman B, Ha I, Ruvkun G. Posttranscriptional regulation of the heterochronic gene lin-14 by lin-4 mediates temporal pattern formation in C. elegans. Cell 1993 12 3;75(5):855–862. 8252622 10.1016/0092-8674(93)90530-4

[5] Lee Y, Jeon K, Lee JT, Kim S, Kim VN. MicroRNA maturation: stepwise processing and subcellular localization. EMBO J 2002 9 2;21(17):4663–4670. 12198168 10.1093/emboj/cdf476 PMC126204

[6] Lee Y, Kim M, Han J, Yeom KH, Lee S, Baek SH, et al MicroRNA genes are transcribed by RNA polymerase II. EMBO J 2004 10 13;23(20):4051–4060. 15372072 10.1038/sj.emboj.7600385 PMC524334

[7] Han J, Lee Y, Yeom KH, Kim YK, Jin H, Kim VN. The Drosha-DGCR8 complex in primary microRNA processing. Genes Dev 2004 12 15;18(24):3016–3027. 15574589 10.1101/gad.1262504 PMC535913

[8] Lee Y, Ahn C, Han J, Choi H, Kim J, Yim J, et al The nuclear RNase III Drosha initiates microRNA processing. Nature 2003 9 25;425(6956):415–419. 14508493 10.1038/nature01957

[9] Bohnsack MT, Czaplinski K, Gorlich D. Exportin 5 is a RanGTP-dependent dsRNA-binding protein that mediates nuclear export of pre-miRNAs. RNA 2004 2;10(2):185–191. 14730017 10.1261/rna.5167604 PMC1370530

[10] Ketting RF, Fischer SE, Bernstein E, Sijen T, Hannon GJ, Plasterk RH. Dicer functions in RNA interference and in synthesis of small RNA involved in developmental timing in C. elegans. Genes Dev 2001 10 15;15(20):2654–2659. 11641272 10.1101/gad.927801 PMC312808

[11] Bartel DP. MicroRNAs: target recognition and regulatory functions. Cell 2009 1 23;136(2):215–233. 10.1016/j.cell.2009.01.002 19167326 PMC3794896

[12] Ha M, Kim VN. Regulation of microRNA biogenesis. Nat Rev Mol Cell Biol 2014 8;15(8):509–524. 10.1038/nrm3838 25027649

[13] Im HI, Kenny PJ. MicroRNAs in neuronal function and dysfunction. Trends Neurosci 2012 5;35(5):325–334. 10.1016/j.tins.2012.01.004 22436491 PMC3565236

[14] Lujambio A, Lowe SW. The microcosmos of cancer. Nature 2012;482(7385):347–355. 10.1038/nature10888 22337054 PMC3509753

[15] Recht MI, Fourmy D, Blanchard SC, Dahlquist KD, Puglisi JD. RNA sequence determinants for aminoglycoside binding to an A-site rRNA model oligonucleotide. J Mol Biol 1996 10 4;262:421–436. 8893854 10.1006/jmbi.1996.0526

[16] Lynch SR, Puglisi JD. Structural Origins of Aminoglycoside Specificity for Prokaryotic Ribosomes. J Mol Biol 2001;306(5):1037–1058. 11237617 10.1006/jmbi.2000.4420

[17] Kumar S, Xue L, Arya DP. Neomycin-Neomycin Dimer: An All-Carbohydrate Scaffold with High Affinity for AT-Rich DNA Duplexes. J Am Chem Soc 2011;133:7361–7375. 10.1021/ja108118v 21524066 PMC3641821

[18] Willis B, Arya DP. Triple Recognition of B-DNA. Bioorg. Med. Chem. Lett. 2009;19:4974–4979. 10.1016/j.bmcl.2009.07.079 19651510

[19] Hamilton PL, Arya DP. Natural product DNA major groove binders. Nat Prod Rep 2012 2 17;29(2):134–143. 10.1039/c1np00054c 22183179

[20] Willis B, Arya DP. An expanding view of aminoglycoside-nucleic acid recognition. Adv Carbohydr Chem Biochem 2006;60:251–302. 16750445 10.1016/S0065-2318(06)60006-1

[21] Shaw NN, Xi H, Arya DP. Molecular recognition of a DNA:RNA hybrid: sub-nanomolar binding by a neomycin-methidium conjugate. Bioorg Med Chem Lett 2008 7 15;18(14):4142–4145. 10.1016/j.bmcl.2008.05.090 18573660

[22] Shaw NN, Arya DP. Recognition of the unique structure of DNA:RNA hybrids. Biochimie 2008 7;90(7):1026–1039. 10.1016/j.biochi.2008.04.011 18486626

[23] King A, Watkins D, Kumar S, Ranjan N, Gong C, Whitlock J, et al Characterization of Ribosomal Binding and Antibacterial Activities Using Two Orthogonal High Throughput Capable Screens. Antimicrob Agents Chemother 2013 7 15. 10.1128/AAC.00671-13 PMC3811423 23856777

[24] Watkins D, Norris FA, Kumar S, Arya DP. A fluorescence-based screen for ribosome binding antibiotics. Anal. Biochem. 2013 3 15;434(2):300–307. 10.1016/j.ab.2012.12.003 23262284 PMC4048832

[25] Ranjan N, Davis E, Xue L, Arya DP. Dual recognition of the human telomeric G-quadruplex by a neomycin-anthraquinone conjugate. Chem Commun 2013;49(51):5796–5798. 10.1039/c3cc42721h PMC3977216 23698792

[26] Watkins D, Ranjan N, Kumar S, Gong C, Arya DP. An assay for human telomeric G-quadruplex DNA binding drugs. Bioorg Med Chem Lett 2013 12 15;23(24):6695–6699. 10.1016/j.bmcl.2013.10.030 24246738 PMC4112084

[27] Ranjan N, Arya DP. Targeting C-myc G-quadruplex: dual recognition by aminosugar-bisbenzimidazoles with varying linker lengths. Molecules 2013 11 18;18(11):14228–14240. 10.3390/molecules181114228 24252993 PMC6270413

[28] Ranjan N, Andreasen KF, Kumar S, Hyde-Volpe D, Arya DP. Aminoglycoside Binding to Oxytricha nova Telomeric DNA. Biochemistry 2010 10 22;49:9891–9903. 10.1021/bi101517e 20886815 PMC3641841

[29] Xue LA, Ranjan N, Arya DP. Synthesis and Spectroscopic Studies of the Aminoglycoside (Neomycin)-Perylene Conjugate Binding to Human Telomeric DNA. Biochemistry-Us 2011 4 12;50(14):2838–2849. 10.1021/bi1017304 21329360

[30] Arya DP. New approaches toward recognition of nucleic Acid triple helices. Acc Chem Res 2011 2 15;44(2):134–146. 10.1021/ar100113q 21073199 PMC3977315

[31] Xi H, Kumar S, Dosen-Micovic L, Arya DP. Calorimetric and spectroscopic studies of aminoglycoside binding to AT-rich DNA triple helices. Biochimie 2010 5;92(5):514–529. 10.1016/j.biochi.2010.02.004 20167243 PMC3977217

[32] Arya DP, Micovic L, Charles I, Coffee RL Jr., Willis B, Xue L. Neomycin binding to Watson-Hoogsteen (W-H) DNA triplex groove: a model. J Am Chem Soc 2003 4 2;125(13):3733–3744. 12656603 10.1021/ja027765m

[33] Arya DP, Willis B. Reaching into the major groove of B-DNA: Synthesis and nucleic acid binding of a neomycin-Hoechst 33258 conjugate. J Am Chem Soc 2003 10 15;125(41):12398–12399. 14531669 10.1021/ja036742k

[34] Arya DP, Xue L, Willis B. Aminoglycoside Preference is for A-form Nucleic Acids, Not Just RNA: Results from a Competition Dialysis Study. J Am Chem Soc 2003:in press. 10.1021/ja035117c 12926918

[35] Arya DP, Coffee RL Jr., Charles I. Neomycin-Induced Hybrid Triplex Formation. J Am Chem Soc 2001;123:11093–11094. 11686727 10.1021/ja016481j

[36] Arya DP, Coffee RL Jr, Willis B, Abramovitch AI. Aminoglycoside-nucleic acid interactions: remarkable stabilization of DNA and RNA triple helices by neomycin. J Am Chem Soc 2001 6 13;123(23):5385–5395. 11389616 10.1021/ja003052x

[37] Ranjan N, Kumar S, Watkins D, Wang D, Appella DH, Arya DP. Recognition of HIV-TAR RNA unsing neomycin-benzimidazole conjugates. Bioorg Med Chem Lett 2013;20:5689–5693. 10.1016/j.bmcl.2013.08.014 PMC4048829 24012122

[38] Kumar S, Kellish P, Robinson WE, Wang D, Appella DH, Arya DP. Click Dimers To Target HIV TAR RNA Conformation. Biochemistry 2012;51(Copyright (C) 2012 American Chemical Society (ACS). All Rights Reserved.):2331–2347. 10.1021/bi201657k 22339203 PMC3673543

[39] Maiti M, Nauwelaerts K, Herdewijn P. Pre-microRNA binding aminoglycosides and antitumor drugs as inhibitors of Dicer catalyzed microRNA processing. Bioorg Med Chem Lett 2012 2 15;22(4):1709–1711. 10.1016/j.bmcl.2011.12.103 22257890

[40] Velagapudi SP, Seedhouse SJ, French J, Disney MD. Defining the RNA Internal Loops Preferred by Benzimidazole Derivatives via 2D Combinatorial Screening and Computational Analysis. J Am Chem Soc 2011 07/06; 2011/12;133(26):10111–10118. 10.1021/ja200212b 21604752 PMC3126894

[41] Velagapudi SP, Gallo SM, Disney MD. Sequence-based design of bioactive small molecules that target precursor microRNAs. Nat Chem Biol 2014 4;10(4):291–297. 10.1038/nchembio.1452 24509821 PMC3962094

[42] Xi H, Davis E, Ranjan N, Xue L, Hyde-Volpe D, Arya DP. Thermodynamics of nucleic Acid "shape readout" by an aminosugar. Biochemistry 2011 10 25;50(42):9088–9113. 10.1021/bi201077h 21863895 PMC3673541

[43] Jiang L, Watkins D, Jin Y, Gong C, King A, Washington AZ, et al Rapid Synthesis, RNA Binding, and Antibacterial Screening of a Peptidic-Aminosugar (PA) Library. ACS Chem Biol 2015;10(5):1278–1289. 10.1021/cb5010367 25706406 PMC4837463

[44] Willis B, Arya DP. Recognition of B-DNA by neomycin—Hoechst 33258 conjugates. Biochemistry 2006 8 29;45(34):10217–10232. 16922497 10.1021/bi0609265

[45] Livak KJ, Schmittgen TD. Analysis of relative gene expression data using real-time quantitative PCR and the 2(-Delta Delta C(T)) Method. Methods 2001, 25, 402–408. 11846609 10.1006/meth.2001.1262

[46] Lin S, Gregory RI. MicroRNA biogenesis pathways in cancer. Nat Rev Cancer 2015 6;15(6):321–333. 10.1038/nrc3932 25998712 PMC4859809

[47] Friedrich K, Woolley P. Electrostatic Potential of Macromolecules Measured by pKa Shift of a Fluorophore. Eur J Biochem 1988;173:227–231. 2833391 10.1111/j.1432-1033.1988.tb13988.x

[48] Alvarez-Pez J, Ballesteros L, Talavera E, Yguerabide J. Fluorescein excited-state proton exchange reactions: Nanosecond emission kinetics and correlation with steady-state fluorescence intensity. Journal of Physical Chemistry a 2001 7 5;105(26):6320–6332.

[49] Lavis LD, Rutkoski TJ, Raines RT. Tuning the pKa of fluorescein to optimize binding assays. Anal Chem 2007;79:6775–6782. 17672523 10.1021/ac070907g PMC2868592

[50] Knemeyer JP, Marme N, Sauer M. Probes for detection of specific DNA sequences at the single-molecule level. Anal Chem 2000 8 15;72(16):3717–3724. 10959954 10.1021/ac000024o

[51] Marras SA, Kramer FR, Tyagi S. Efficiencies of fluorescence resonance energy transfer and contact-mediated quenching in oligonucleotide probes. Nucleic Acids Res 2002 11 1;30(21):e122 12409481 10.1093/nar/gnf121 PMC135848

[52] Ranasinghe RT, Brown T. Fluorescence based strategies for genetic analysis. Chem Commun (Camb) 2005 11 28;(44)(44):5487–5502. 16358042 10.1039/b509522k

[53] Sjoback R, Nygren J, Kubista M. Characterization of Fluorescein-Oligonucleotide Conjugates and Measurement of Local Electrostatic Potential. Biopolymers 1998;46:445–453. 9838871 10.1002/(SICI)1097-0282(199812)46:7<445::AID-BIP2>3.0.CO;2-5

[54] Watkins D, Norris FA, Kumar S, Arya DP. A fluorescence-based screen for ribosome binding antibiotics. Anal Biochem 2013 3 15;434(2):300–307. 10.1016/j.ab.2012.12.003 23262284 PMC4048832

[55] Zhang JH, Chung TD, Oldenburg KR. A Simple Statistical Parameter for Use in Evaluation and Validation of High Throughput Screening Assays. J Biomol Screen 1999;4(2):67–73. 10838414 10.1177/108705719900400206

[56] Kozomara A, Griffiths-Jones S. miRBase: annotating high confidence microRNAs using deep sequencing data. Nucleic Acids Res 2014 1;42(Database issue):D68–73. 10.1093/nar/gkt1181 24275495 PMC3965103

[57] Kozomara A, Griffiths-Jones S. miRBase: integrating microRNA annotation and deep-sequencing data. Nucleic Acids Res 2011 1;39(Database issue):D152–7. 10.1093/nar/gkq1027 21037258 PMC3013655

[58] Griffiths-Jones S, Saini HK, van Dongen S, Enright AJ. miRBase: tools for microRNA genomics. Nucleic Acids Res 2007;36(Database):D154–D158. 17991681 10.1093/nar/gkm952 PMC2238936

[59] Griffiths-Jones S. miRBase: microRNA sequences, targets and gene nomenclature. Nucleic Acids Res 2006;34(90001):D140–D144. 16381832 10.1093/nar/gkj112 PMC1347474

[60] Griffiths-Jones S. The microRNA Registry. Nucleic Acids Res 2004;32(90001):109D–111. 10.1093/nar/gkh023 PMC308757 14681370

[61] Trott O, Olson AJ. AutoDock Vina: Improving the speed and accuracy of docking with a new scoring function, efficient optimization, and multithreading. J Comput Chem 2010 6 4;31:455–461. 10.1002/jcc.21334 19499576 PMC3041641

