# Supplementary material for: A pH Sensitive High-Throughput Assay for miRNA Binding of a Peptide-Aminoglycoside (PA) Library
Source: PLoS One. 2015 Dec 11;10(12):e0144251. doi: 10.1371/journal.pone.0144251 (PMC4699463; doi:10.1371/journal.pone.0144251)
Supplement: S1 Table — (DOCX) [file pone.0144251.s001.docx]

S1 Table. DPA numbered compounds with percent binding of neomycin

| DPA No. |  | hsa-miR 142 | | | hsa-miR 335 | | | hsa-miR 504 | | | Pre hsa-miR 504 | | |
| --- | --- | --- | --- | --- | --- | --- | --- | --- | --- | --- | --- | --- | --- |
| Neomycin | **Neo** |  |  |  | 100 |  |  | 100 |  |  | 100 |  |  |
| DPA1101 | **NeoβA** | 56 | ± | 7 | 72 | ± | 14 | 80 | ± | 4 | 82 | ± | 2 |
| DPA1102 | **NeoR** | 96 | ± | 16 | 94 | ± | 3 | 114 | ± | 2 | 101 | ± | 5 |
| DPA1103 | **NeoN** | 66 | ± | 16 | 80 | ± | 6 | 87 | ± | 9 | 89 | ± | 14 |
| DPA1104 | **NeoD** | 40 | ± | 1 | 38 | ± | 5 | 55 | ± | 4 | 45 | ± | 6 |
| DPA1105 | **NeoH** | 47 | ± | 7 | 68 | ± | 10 | 95 | ± | 2 | 74 | ± | 9 |
| DPA1106 | **NeoL** | 38 | ± | 2 | 65 | ± | 0 | 82 | ± | 8 | 77 | ± | 8 |
| DPA1107 | **NeoF** | 36 | ± | 2 | 69 | ± | 5 | 67 | ± | 1 | 72 | ± | 7 |
| DPA1108 | **NeoP** | 19 | ± | 1 | 20 | ± | 4 | 76 | ± | 1 | 76 | ± | 6 |
| DPA1109 | **NeoS** | 13 | ± | 3 | 78 | ± | 12 | 62 | ± | 1 | 73 | ± | 11 |
| DPA1110 | **NeoT** | 37 | ± | 1 | 75 | ± | 3 | 86 | ± | 11 | 69 | ± | 4 |
| DPA1111 | **NeoY** | 45 | ± | 18 | 68 | ± | 3 | 53 | ± | 10 | 30 | ± | 5 |
| DPA1112 | **NeoV** | 11 | ± | 6 | 51 | ± | 1 | 88 | ± | 1 | 27 | ± | 5 |
| DPA1113 | **NeoC** | 38 | ± | 15 | 72 | ± | 8 | 64 | ± | 8 | 44 | ± | 7 |
| DPA1114 | **NeoW** | 47 | ± | 7 | 73 | ± | 2 | 67 | ± | 1 | 54 | ± | 2 |
| DPA1115 | **NeoK** | 77 | ± | 9 | 81 | ± | 6 | 86 | ± | 10 | 70 | ± | 4 |
| DPA1116 | **NeoβAβA** | 81 | ± | 7 | 84 | ± | 5 | 78 | ± | 7 | 63 | ± | 5 |
| DPA1117 | **NeoRβA** | 59 | ± | 38 | 87 | ± | 6 | 74 | ± | 3 | 95 | ± | 10 |
| DPA1118 | **NeoNβA** | 15 | ± | 7 | 62 | ± | 24 | 71 | ± | 12 | 61 | ± | 10 |
| DPA1119 | **NeoDβA** | 15 | ± | 3 | 66 | ± | 16 | 41 | ± | 3 | 31 | ± | 2 |
| DPA1120 | **NeoHβA** | 27 | ± | 2 | 72 | ± | 3 | 61 | ± | 5 | 72 | ± | 4 |
| DPA1121 | **NeoLβA** | 52 | ± | 5 | 30 | ± | 1 | 48 | ± | 2 | 69 | ± | 3 |
| DPA1122 | **NeoFβA** | 55 | ± | 4 | 49 | ± | 1 | 48 | ± | 1 | 60 | ± | 4 |
| DPA1123 | **NeoPβA** | 53 | ± | 11 | 44 | ± | 4 | 93 | ± | 4 | 65 | ± | 7 |
| DPA1124 | **NeoSβA** | 30 | ± | 6 | 73 | ± | 11 | 93 | ± | 1 | 56 | ± | 5 |
| DPA1125 | **NeoTβA** | 22 | ± | 7 | 52 | ± | 16 | 89 | ± | 14 | 57 | ± | 11 |
| DPA1126 | **NeoYβA** | 29 | ± | 1 | 73 | ± | 1 | 35 | ± | 1 | 51 | ± | 7 |
| DPA1127 | **NeoVβA** | 51 | ± | 3 | 45 | ± | 19 | 39 | ± | 3 | 53 | ± | 10 |
| DPA1128 | **NeoCβA** | 65 | ± | 2 | 75 | ± | 12 | 41 | ± | 3 | 44 | ± | 3 |
| DPA1129 | **NeoWβA** | 28 | ± | 6 | 56 | ± | 0 | 60 | ± | 8 | 53 | ± | 1 |
| DPA1130 | **Neo****βAR** | 75 | ± | 1 | 72 | ± | 13 | 79 | ± | 7 | 75 | ± | 19 |
| DPA1131 | **NeoRR** | 71 | ± | 1 | 76 | ± | 17 | 85 | ± | 6 | 89 | ± | 17 |
| DPA1132 | **NeoNR** | 78 | ± | 3 | 65 | ± | 3 | 93 | ± | 5 | 74 | ± | 14 |
| DPA1133 | **NeoDR** | 36 | ± | 0 | 43 | ± | 7 | 79 | ± | 2 | 47 | ± | 4 |
| DPA1134 | **NeoHR** | 69 | ± | 7 | 55 | ± | 2 | 90 | ± | 8 | 71 | ± | 0 |
| DPA1135 | **NeoLR** | 46 | ± | 15 | 65 | ± | 13 | 78 | ± | 8 | 28 | ± | 8 |
| DPA1136 | **NeoFR** | 42 | ± | 5 | 57 | ± | 7 | 78 | ± | 3 | 33 | ± | 9 |
| DPA1137 | **NeoPR** | 28 | ± | 17 | 55 | ± | 11 | 78 | ± | 2 | 48 | ± | 3 |
| DPA1138 | **NeoSR** | 73 | ± | 2 | 75 | ± | 4 | 89 | ± | 2 | 56 | ± | 3 |
| DPA1139 | **NeoTR** | 36 | ± | 6 | 81 | ± | 2 | 73 | ± | 4 | 59 | ± | 3 |
| DPA1140 | **NeoYR** | 57 | ± | 2 | 83 | ± | 19 | 77 | ± | 2 | 66 | ± | 5 |
| DPA1141 | **NeoVR** | 55 | ± | 6 | 68 | ± | 2 | 89 | ± | 3 | 71 | ± | 2 |
| DPA1142 | **NeoCR** | 62 | ± | 4 | 60 | ± | 22 | 62 | ± | 6 | 67 | ± | 3 |
| DPA1143 | **NeoWR** | 57 | ± | 5 | 66 | ± | 1 | 70 | ± | 3 | 65 | ± | 1 |
| DPA1144 | **NeoβAN** | 65 | ± | 3 | 50 | ± | 2 | 87 | ± | 14 | 55 | ± | 4 |
| DPA1145 | **NeoRN** | 44 | ± | 11 | 80 | ± | 5 | 80 | ± | 6 | 66 | ± | 10 |
| DPA1146 | **NeoNN** | 27 | ± | 9 | 61 | ± | 2 | 63 | ± | 3 | 48 | ± | 4 |
| DPA1147 | **NeoDN** | 9 | ± | 7 | 12 | ± | 14 | 25 | ± | 1 | 70 | ± | 13 |
| DPA1148 | **NeoHN** | 32 | ± | 3 | 88 | ± | 3 | 61 | ± | 4 | 82 | ± | 1 |
| DPA1149 | **NeoLN** | 60 | ± | 0 | 24 | ± | 14 | 73 | ± | 14 | 63 | ± | 4 |
| DPA1150 | **NeoFN** | 59 | ± | 7 | 54 | ± | 8 | 63 | ± | 8 | 79 | ± | 11 |
| DPA1151 | **NeoPN** | 67 | ± | 0 | 45 | ± | 14 | 78 | ± | 5 | 87 | ± | 4 |
| DPA1152 | **NeoSN** | 71 | ± | 5 | 58 | ± | 6 | 65 | ± | 8 | 79 | ± | 3 |
| DPA1153 | **NeoTN** | 66 | ± | 1 | 54 | ± | 11 | 77 | ± | 9 | 88 | ± | 17 |
| DPA1154 | **NeoYN** | 15 | ± | 13 | 36 | ± | 18 | 61 | ± | 11 | 81 | ± | 6 |
| DPA1155 | **NeoVN** | 25 | ± | 6 | 31 | ± | 14 | 59 | ± | 3 | 79 | ± | 12 |
| DPA1156 | **NeoCN** | 23 | ± | 4 | 24 | ± | 16 | 35 | ± | 2 | 59 | ± | 7 |
| DPA1157 | **NeoWN** | 49 | ± | 12 | 44 | ± | 7 | 65 | ± | 7 | 80 | ± | 13 |
| DPA1158 | **NeoβAD** | 42 | ± | 8 | 23 | ± | 2 | 31 | ± | 4 | 63 | ± | 7 |
| DPA1159 | **NeoRD** | 74 | ± | 2 | 59 | ± | 1 | 60 | ± | 0 | 84 | ± | 18 |
| DPA1160 | **NeoND** | 28 | ± | 11 | 39 | ± | 2 | 26 | ± | 2 | 68 | ± | 6 |
| DPA1161 | **NeoDD** | 51 | ± | 10 | 42 | ± | 9 | 23 | ± | 1 | 74 | ± | 9 |
| DPA1162 | **NeoHD** | 37 | ± | 2 | 27 | ± | 7 | 9 | ± | 2 | 37 | ± | 8 |
| DPA1163 | **NeoLD** | 15 | ± | 4 | 18 | ± | 17 | 13 | ± | 2 | 38 | ± | 17 |
| DPA1164 | **NeoFD** | 18 | ± | 4 | 13 | ± | 6 | 12 | ± | 3 | 48 | ± | 5 |
| DPA1165 | **NeoPD** | 16 | ± | 0 | 15 | ± | 8 | 48 | ± | 7 | 40 | ± | 9 |
| DPA1166 | **NeoSD** | 24 | ± | 10 | 6 | ± | 13 | 64 | ± | 3 | 55 | ± | 10 |
| DPA1167 | **NeoTD** | 19 | ± | 5 | 14 | ± | 11 | 34 | ± | 9 | 54 | ± | 5 |
| DPA1168 | **NeoYD** | 12 | ± | 8 | 27 | ± | 3 | 68 | ± | 1 | 43 | ± | 9 |
| DPA1169 | **NeoVD** | 20 | ± | 6 | 20 | ± | 9 | 56 | ± | 2 | 42 | ± | 4 |
| DPA1170 | **NeoCD** | 21 | ± | 7 | 21 | ± | 7 | 26 | ± | 6 | 69 | ± | 2 |
| DPA1171 | **NeoWD** | 15 | ± | 2 | 14 | ± | 9 | 49 | ± | 8 | 34 | ± | 2 |
| DPA1172 | **NeoβAH** | 55 | ± | 8 | 48 | ± | 5 | 86 | ± | 3 | 66 | ± | 8 |
| DPA1173 | **NeoRH** | 60 | ± | 6 | 50 | ± | 5 | 121 | ± | 12 | 102 | ± | 1 |
| DPA1174 | **NeoNH** | 51 | ± | 2 | 65 | ± | 4 | 114 | ± | 6 | 63 | ± | 11 |
| DPA1175 | **NeoDH** | 23 | ± | 10 | 42 | ± | 1 | 93 | ± | 14 | 53 | ± | 3 |
| DPA1176 | **NeoHH** | 47 | ± | 11 | 56 | ± | 4 | 75 | ± | 12 | 75 | ± | 4 |
| DPA1177 | **NeoLH** | 30 | ± | 15 | 40 | ± | 9 | 104 | ± | 2 | 44 | ± | 12 |
| DPA1178 | **NeoFH** | 35 | ± | 3 | 46 | ± | 0 | 88 | ± | 10 | 42 | ± | 1 |
| DPA1179 | **NeoPH** | 30 | ± | 4 | 50 | ± | 1 | 94 | ± | 4 | 22 | ± | 3 |
| DPA1180 | **NeoSH** | 55 | ± | 4 | 43 | ± | 7 | 103 | ± | 20 | 71 | ± | 10 |
| DPA1181 | **NeoTH** | 65 | ± | 10 | 43 | ± | 3 | 90 | ± | 2 | 95 | ± | 12 |
| DPA1182 | **NeoYH** | 34 | ± | 4 | 41 | ± | 4 | 79 | ± | 15 | 82 | ± | 2 |
| DPA1183 | **NeoVH** | 40 | ± | 13 | 41 | ± | 2 | 84 | ± | 2 | 75 | ± | 9 |
| DPA1184 | **NeoCH** | 26 | ± | 16 | 41 | ± | 4 | 72 | ± | 14 | 71 | ± | 3 |
| DPA1185 | **NeoWH** | 36 | ± | 1 | 41 | ± | 2 | 60 | ± | 1 | 70 | ± | 7 |
| DPA1186 | **NeoβAL** | 40 | ± | 5 | 43 | ± | 4 | 46 | ± | 5 | 67 | ± | 1 |
| DPA1187 | **NeoRL** | 55 | ± | 6 | 54 | ± | 6 | 62 | ± | 4 | 84 | ± | 6 |
| DPA1188 | **NeoNL** | 39 | ± | 2 | 37 | ± | 12 | 98 | ± | 18 | 60 | ± | 9 |
| DPA1189 | **NeoDL** | 2 | ± | 4 | -1 | ± | 11 | 36 | ± | 4 | 28 | ± | 3 |
| DPA1190 | **NeoHL** | 35 | ± | 8 | 45 | ± | 8 | 89 | ± | 12 | 71 | ± | 6 |
| DPA1191 | **NeoLL** | 28 | ± | 8 | 5 | ± | 6 | 79 | ± | 9 | 46 | ± | 2 |
| DPA1192 | **NeoFL** | 32 | ± | 3 | 22 | ± | 9 | 95 | ± | 13 | 50 | ± | 3 |
| DPA1193 | **NeoPL** | 56 | ± | 5 | 49 | ± | 7 | 94 | ± | 5 | 41 | ± | 1 |
| DPA1194 | **NeoSL** | 67 | ± | 6 | 40 | ± | 2 | 57 | ± | 2 | 64 | ± | 6 |
| DPA1195 | **NeoTL** | 74 | ± | 3 | 47 | ± | 9 | 96 | ± | 16 | 64 | ± | 11 |
| DPA1196 | **NeoYL** | 73 | ± | 3 | 35 | ± | 2 | 42 | ± | 13 | 59 | ± | 3 |
| DPA1197 | **NeoVL** | 66 | ± | 1 | 26 | ± | 8 | 87 | ± | 12 | 31 | ± | 3 |
| DPA1198 | **NeoCL** | 64 | ± | 2 | 18 | ± | 2 | 47 | ± | 13 | 29 | ± | 3 |
| DPA1199 | **NeoWL** | 38 | ± | 0 | 8 | ± | 8 | 78 | ± | 15 | 52 | ± | 1 |
| DPA1200 | **NeoβAF** | 66 | ± | 3 | 38 | ± | 14 | 37 | ± | 10 | 71 | ± | 9 |
| DPA1201 | **NeoRF** | 68 | ± | 1 | 40 | ± | 3 | 82 | ± | 16 | 88 | ± | 3 |
| DPA1202 | **NeoNF** | 78 | ± | 2 | 27 | ± | 10 | 83 | ± | 0 | 57 | ± | 10 |
| DPA1203 | **NeoDF** | 49 | ± | 1 | 25 | ± | 6 | 46 | ± | 14 | 28 | ± | 5 |
| DPA1204 | **NeoHF** | 74 | ± | 1 | 52 | ± | 19 | 81 | ± | 17 | 74 | ± | 10 |
| DPA1205 | **NeoLF** | 61 | ± | 8 | 35 | ± | 10 | 71 | ± | 1 | 48 | ± | 3 |
| DPA1206 | **NeoFF** | 61 | ± | 4 | 43 | ± | 16 | 73 | ± | 1 | 68 | ± | 16 |
| DPA1207 | **NeoPF** | 56 | ± | 0 | 43 | ± | 16 | 64 | ± | 4 | 55 | ± | 5 |
| DPA1208 | **NeoSF** | 67 | ± | 4 | 50 | ± | 15 | 78 | ± | 5 | 62 | ± | 14 |
| DPA1209 | **NeoTF** | 65 | ± | 4 | 34 | ± | 12 | 69 | ± | 10 | 68 | ± | 1 |
| DPA1210 | **NeoYF** | 53 | ± | 4 | 31 | ± | 5 | 74 | ± | 7 | 59 | ± | 2 |
| DPA1211 | **NeoVF** | 58 | ± | 4 | 31 | ± | 8 | 76 | ± | 11 | 49 | ± | 10 |
| DPA1212 | **NeoCF** | 44 | ± | 3 | 26 | ± | 12 | 51 | ± | 10 | 62 | ± | 7 |
| DPA1213 | **NeoWF** | 42 | ± | 7 | 0 | ± | 7 | 67 | ± | 0 | 40 | ± | 9 |
| DPA1214 | **NeoβAP** | 49 | ± | 1 | 44 | ± | 7 | 74 | ± | 7 | 43 | ± | 16 |
| DPA1215 | **NeoRP** | 81 | ± | 5 | 67 | ± | 11 | 83 | ± | 16 | 51 | ± | 8 |
| DPA1216 | **NeoNP** | 74 | ± | 4 | 48 | ± | 11 | 79 | ± | 17 | 36 | ± | 7 |
| DPA1217 | **NeoDP** | 50 | ± | 5 | 23 | ± | 2 | 31 | ± | 10 | 3 | ± | 9 |
| DPA1218 | **NeoHP** | 74 | ± | 0 | 59 | ± | 7 | 63 | ± | 2 | 107 | ± | 17 |
| DPA1219 | **NeoLP** | 58 | ± | 1 | 38 | ± | 1 | 50 | ± | 4 | 85 | ± | 14 |
| DPA1220 | **NeoFP** | 65 | ± | 4 | 54 | ± | 2 | 53 | ± | 1 | 104 | ± | 14 |
| DPA1221 | **NeoPP** | 63 | ± | 2 | 45 | ± | 8 | 88 | ± | 7 | 84 | ± | 3 |
| DPA1222 | **NeoSP** | 68 | ± | 4 | 42 | ± | 2 | 69 | ± | 17 | 98 | ± | 11 |
| DPA1223 | **NeoTP** | 50 | ± | 4 | 34 | ± | 4 | 72 | ± | 13 | 95 | ± | 16 |
| DPA1224 | **NeoYP** | 60 | ± | 7 | 28 | ± | 7 | 98 | ± | 10 | 74 | ± | 16 |
| DPA1225 | **NeoVP** | 53 | ± | 4 | 29 | ± | 6 | 117 | ± | 1 | 85 | ± | 14 |
| DPA1226 | **NeoCP** | 49 | ± | 9 | 94 | ± | 16 | 60 | ± | 12 | 83 | ± | 5 |
| DPA1227 | **NeoWP** | 48 | ± | 3 | 38 | ± | 14 | 64 | ± | 12 | 91 | ± | 11 |
| DPA1228 | **NeoβAS** | 88 | ± | 5 | 60 | ± | 4 | 122 | ± | 12 | 93 | ± | 6 |
| DPA1229 | **NeoRS** | 90 | ± | 4 | 69 | ± | 6 | 87 | ± | 11 | 118 | ± | 5 |
| DPA1230 | **NeoNS** | 87 | ± | 4 | 64 | ± | 1 | 127 | ± | 14 | 97 | ± | 9 |
| DPA1231 | **NeoDS** | 54 | ± | 0 | 33 | ± | 0 | 93 | ± | 6 | 71 | ± | 10 |
| DPA1232 | **NeoHS** | 76 | ± | 4 | 61 | ± | 2 | 99 | ± | 11 | 94 | ± | 9 |
| DPA1233 | **NeoLS** | 70 | ± | 6 | 56 | ± | 4 | 107 | ± | 1 | 92 | ± | 3 |
| DPA1234 | **NeoFS** | 64 | ± | 9 | 44 | ± | 9 | 115 | ± | 3 | 93 | ± | 11 |
| DPA1235 | **NeoPS** | 57 | ± | 3 | 43 | ± | 9 | 105 | ± | 16 | 74 | ± | 13 |
| DPA1236 | **NeoSS** | 67 | ± | 3 | 57 | ± | 3 | 106 | ± | 4 | 94 | ± | 7 |
| DPA1237 | **NeoTS** | 70 | ± | 5 | 43 | ± | 9 | 102 | ± | 5 | 72 | ± | 15 |
| DPA1238 | **NeoYS** | 75 | ± | 3 | 45 | ± | 7 | 104 | ± | 8 | 78 | ± | 11 |
| DPA1239 | **NeoVS** | 108 | ± | 6 | 70 | ± | 9 | 107 | ± | 14 | 89 | ± | 7 |
| DPA1240 | **NeoCS** | 125 | ± | 3 | 72 | ± | 6 | 84 | ± | 15 | 71 | ± | 6 |
| DPA1241 | **NeoWS** | 85 | ± | 5 | 37 | ± | 3 | 62 | ± | 10 | 58 | ± | 0 |
| DPA1242 | **NeoβAT** | 104 | ± | 9 | 69 | ± | 17 | 86 | ± | 15 | 62 | ± | 6 |
| DPA1243 | **NeoRT** | 72 | ± | 3 | 68 | ± | 2 | 92 | ± | 2 | 63 | ± | 8 |
| DPA1244 | **NeoNT** | 67 | ± | 11 | 42 | ± | 6 | 91 | ± | 15 | 92 | ± | 2 |
| DPA1245 | **NeoDT** | 54 | ± | 15 | 29 | ± | 15 | 59 | ± | 5 | 38 | ± | 7 |
| DPA1246 | **NeoHT** | 55 | ± | 0 | 54 | ± | 13 | 94 | ± | 1 | 86 | ± | 7 |
| DPA1247 | **NeoLT** | 61 | ± | 3 | 51 | ± | 8 | 79 | ± | 2 | 92 | ± | 10 |
| DPA1248 | **NeoFT** | 79 | ± | 9 | 64 | ± | 13 | 94 | ± | 11 | 93 | ± | 4 |
| DPA1249 | **NeoPT** | 94 | ± | 8 | 81 | ± | 13 | 92 | ± | 8 | 87 | ± | 2 |
| DPA1250 | **NeoST** | 93 | ± | 4 | 87 | ± | 4 | 90 | ± | 5 | 88 | ± | 2 |
| DPA1251 | **NeoTT** | 91 | ± | 4 | 88 | ± | 4 | 76 | ± | 5 | 78 | ± | 4 |
| DPA1252 | **NeoYT** | 70 | ± | 3 | 69 | ± | 15 | 65 | ± | 4 | 84 | ± | 2 |
| DPA1253 | **NeoVT** | 82 | ± | 1 | 73 | ± | 8 | 58 | ± | 3 | 81 | ± | 15 |
| DPA1254 | **NeoCT** | 56 | ± | 9 | 44 | ± | 7 | 25 | ± | 2 | 58 | ± | 0 |
| DPA1255 | **NeoWT** | 48 | ± | 1 | 51 | ± | 2 | 74 | ± | 6 | 68 | ± | 5 |
| DPA1256 | **NeoβAY** | 76 | ± | 3 | 57 | ± | 4 | 76 | ± | 15 | 84 | ± | 1 |
| DPA1257 | **NeoRY** | 73 | ± | 10 | 72 | ± | 11 | 100 | ± | 3 | 106 | ± | 6 |
| DPA1258 | **NeoNY** | 60 | ± | 6 | 54 | ± | 14 | 74 | ± | 10 | 88 | ± | 11 |
| DPA1259 | **NeoDY** | 41 | ± | 5 | 23 | ± | 10 | 41 | ± | 11 | 47 | ± | 3 |
| DPA1260 | **NeoHY** | 78 | ± | 9 | 51 | ± | 6 | 110 | ± | 12 | 94 | ± | 17 |
| DPA1261 | **NeoLY** | 68 | ± | 7 | 83 | ± | 12 | 58 | ± | 9 | 90 | ± | 10 |
| DPA1262 | **NeoFY** | 69 | ± | 1 | 81 | ± | 14 | 106 | ± | 2 | 92 | ± | 9 |
| DPA1263 | **NeoPY** | 86 | ± | 5 | 75 | ± | 17 | 50 | ± | 8 | 83 | ± | 6 |
| DPA1264 | **NeoSY** | 76 | ± | 4 | 61 | ± | 13 | 99 | ± | 1 | 76 | ± | 10 |
| DPA1265 | **NeoTY** | 67 | ± | 1 | 75 | ± | 1 | 98 | ± | 14 | 63 | ± | 5 |
| DPA1266 | **NeoYY** | 78 | ± | 2 | 70 | ± | 19 | 93 | ± | 1 | 66 | ± | 9 |
| DPA1267 | **NeoVY** | 61 | ± | 1 | 53 | ± | 15 | 87 | ± | 8 | 47 | ± | 5 |
| DPA1268 | **NeoCY** | 49 | ± | 1 | 40 | ± | 11 | 47 | ± | 1 | 25 | ± | 5 |
| DPA1269 | **NeoWY** | 45 | ± | 10 | 29 | ± | 19 | 73 | ± | 8 | 90 | ± | 10 |
| DPA1270 | **NeoβAV** | 58 | ± | 3 | 56 | ± | 9 | 106 | ± | 13 | 73 | ± | 3 |
| DPA1271 | **NeoRV** | 72 | ± | 2 | 66 | ± | 5 | 106 | ± | 10 | 112 | ± | 19 |
| DPA1272 | **NeoNV** | 67 | ± | 2 | 58 | ± | 11 | 87 | ± | 5 | 92 | ± | 13 |
| DPA1273 | **NeoDV** | 36 | ± | 3 | 44 | ± | 12 | 27 | ± | 8 | 61 | ± | 7 |
| DPA1274 | **NeoHV** | 61 | ± | 1 | 79 | ± | 10 | 87 | ± | 12 | 98 | ± | 15 |
| DPA1275 | **NeoLV** | 62 | ± | 1 | 54 | ± | 11 | 94 | ± | 8 | 86 | ± | 13 |
| DPA1276 | **NeoFV** | 57 | ± | 0 | 58 | ± | 9 | 92 | ± | 5 | 97 | ± | 17 |
| DPA1277 | **NeoPV** | 67 | ± | 0 | 62 | ± | 11 | 86 | ± | 3 | 89 | ± | 10 |
| DPA1278 | **NeoSV** | 64 | ± | 8 | 58 | ± | 12 | 95 | ± | 1 | 85 | ± | 4 |
| DPA1279 | **NeoTV** | 60 | ± | 1 | 60 | ± | 15 | 91 | ± | 2 | 86 | ± | 13 |
| DPA1280 | **NeoYV** | 44 | ± | 2 | 59 | ± | 9 | 83 | ± | 10 | 86 | ± | 13 |
| DPA1281 | **NeoVV** | 52 | ± | 1 | 50 | ± | 11 | 82 | ± | 4 | 78 | ± | 15 |
| DPA1282 | **NeoCV** | 46 | ± | 4 | 35 | ± | 10 | 30 | ± | 0 | 71 | ± | 9 |
| DPA1283 | **NeoWV** | 41 | ± | 9 | 27 | ± | 8 | 51 | ± | 8 | 67 | ± | 11 |
| DPA1284 | **NeoβAC** | 52 | ± | 14 | 28 | ± | 8 | 20 | ± | 2 | 52 | ± | 6 |
| DPA1285 | **NeoRC** | 86 | ± | 12 | 99 | ± | 3 | 65 | ± | 3 | 91 | ± | 13 |
| DPA1286 | **NeoNC** | 75 | ± | 13 | 80 | ± | 6 | 61 | ± | 8 | 60 | ± | 13 |
| DPA1287 | **NeoDC** | 40 | ± | 6 | 49 | ± | 10 | 33 | ± | 3 | 37 | ± | 4 |
| DPA1288 | **NeoHC** | 84 | ± | 8 | 81 | ± | 9 | 81 | ± | 8 | 63 | ± | 17 |
| DPA1289 | **NeoLC** | 51 | ± | 5 | 51 | ± | 4 | 55 | ± | 3 | 43 | ± | 1 |
| DPA1290 | **NeoFC** | 55 | ± | 8 | 47 | ± | 4 | 52 | ± | 3 | 33 | ± | 8 |
| DPA1291 | **NeoPC** | 70 | ± | 3 | 53 | ± | 10 | 64 | ± | 8 | 46 | ± | 10 |
| DPA1292 | **NeoSC** | 42 | ± | 1 | 49 | ± | 20 | 68 | ± | 7 | 59 | ± | 15 |
| DPA1293 | **NeoTC** | 56 | ± | 8 | 65 | ± | 9 | 62 | ± | 6 | 58 | ± | 12 |
| DPA1294 | **NeoYC** | 21 | ± | 14 | 66 | ± | 10 | 65 | ± | 2 | 79 | ± | 17 |
| DPA1295 | **NeoVC** | 46 | ± | 7 | 43 | ± | 9 | 45 | ± | 6 | 74 | ± | 14 |
| DPA1296 | **NeoCC** | 55 | ± | 7 | 65 | ± | 6 | 74 | ± | 4 | 52 | ± | 5 |
| DPA1297 | **NeoWC** | 48 | ± | 10 | 51 | ± | 13 | 50 | ± | 5 | 68 | ± | 5 |
| DPA1298 | **NeoβAW** | 49 | ± | 4 | 52 | ± | 9 | 71 | ± | 5 | 59 | ± | 0 |
| DPA1299 | **NeoRW** |  | ± |  |  | ± |  | 84 | ± | 7 | 75 | ± | 6 |
| DPA1300 | **NeoNW** | 41 | ± | 10 | 50 | ± | 10 | 74 | ± | 7 | 73 | ± | 10 |
| DPA1301 | **NeoDW** | 15 | ± | 9 | 13 | ± | 5 | 36 | ± | 2 | 40 | ± | 8 |
| DPA1302 | **NeoHW** | 54 | ± | 8 | 53 | ± | 10 | 66 | ± | 5 | 78 | ± | 10 |
| DPA1303 | **NeoLW** | 34 | ± | 2 | 28 | ± | 11 | 57 | ± | 10 | 61 | ± | 13 |
| DPA1304 | **NeoFW** | 40 | ± | 7 | 42 | ± | 5 | 75 | ± | 15 | 70 | ± | 11 |
| DPA1305 | **NeoPW** | 43 | ± | 5 | 39 | ± | 13 | 58 | ± | 8 | 67 | ± | 18 |
| DPA1306 | **NeoSW** | 49 | ± | 1 | 45 | ± | 5 | 71 | ± | 1 | 71 | ± | 15 |
| DPA1307 | **NeoTW** | 55 | ± | 1 | 47 | ± | 7 | 68 | ± | 0 | 66 | ± | 15 |
| DPA1308 | **NeoYW** | 44 | ± | 7 | 42 | ± | 6 | 60 | ± | 5 | 63 | ± | 9 |
| DPA1309 | **NeoVW** | 43 | ± | 4 | 49 | ± | 3 | 63 | ± | 15 | 67 | ± | 9 |
| DPA1310 | **NeoCW** | 45 | ± | 10 | 52 | ± | 12 | 50 | ± | 15 | 57 | ± | 8 |
| DPA1311 | **NeoWW** | 40 | ± | 8 | 43 | ± | 6 | 58 | ± | 3 | 62 | ± | 9 |
| DPA1312 | **NeoKS** | 65 | ± | 8 | 76 | ± | 2 | 70 | ± | 1 | 83 | ± | 11 |
| DPA1313 | **NeoKT** | 68 | ± | 7 | 70 | ± | 3 | 61 | ± | 2 | 74 | ± | 14 |
| DPA1314 | **NeoKY** | 66 | ± | 8 | 70 | ± | 2 | 56 | ± | 4 | 74 | ± | 10 |
| DPA1315 | **NeoKV** | 67 | ± | 7 | 70 | ± | 3 | 55 | ± | 3 | 62 | ± | 6 |
