# Supplementary material for: A pH Sensitive High-Throughput Assay for miRNA Binding of a Peptide-Aminoglycoside (PA) Library
Source: PLoS One. 2015 Dec 11;10(12):e0144251. doi: 10.1371/journal.pone.0144251 (PMC4699463; doi:10.1371/journal.pone.0144251)
Supplement: S2 Table — (DOCX) [file pone.0144251.s002.docx]

S2 Table. hsa-miR 142 Percent Binding of Neomycin

| Position 2 | Position 1 | | | | | | | | | | | | | | | |
| --- | --- | --- | --- | --- | --- | --- | --- | --- | --- | --- | --- | --- | --- | --- | --- | --- |
|  | *β*A | R | N | D | H | L | F | P | S | T | Y | V | C | W | K | Average  Binding  Position 2 |
| N/A | 56 | 96 | 66 | 40 | 47 | 38 | 36 | 19 | 13 | 37 | 45 | 11 | 38 | 47 | 77 | 44 |
| βA | 81 | 59 | 15 | 15 | 27 | 52 | 55 | 53 | 30 | 22 | 29 | 51 | 65 | 28 |  | 42 |
| R | 75 | 71 | 78 | 36 | 69 | 46 | 42 | 28 | 73 | 36 | 57 | 55 | 62 | 57 |  | 56 |
| N | 65 | 44 | 27 | 9 | 32 | 60 | 59 | 67 | 71 | 66 | 15 | 25 | 23 | 49 |  | 44 |
| D | 42 | 74 | 28 | 51 | 37 | 15 | 18 | 16 | 24 | 19 | 12 | 20 | 21 | 15 |  | 28 |
| H | 55 | 60 | 51 | 23 | 47 | 30 | 35 | 30 | 55 | 65 | 34 | 40 | 26 | 36 |  | 42 |
| L | 40 | 55 | 39 | 2 | 35 | 28 | 32 | 56 | 67 | 74 | 73 | 66 | 64 | 38 |  | 48 |
| F | 66 | 68 | 78 | 49 | 74 | 61 | 61 | 56 | 67 | 65 | 53 | 58 | 44 | 42 |  | 60 |
| P | 49 | 81 | 74 | 50 | 74 | 58 | 65 | 63 | 68 | 50 | 60 | 53 | 49 | 48 |  | 60 |
| S | 88 | 90 | 87 | 54 | 76 | 70 | 64 | 57 | 67 | 70 | 75 | 108 | 125 | 85 | 65 | 79 |
| T | 104 | 72 | 67 | 54 | 55 | 61 | 83 | 94 | 93 | 91 | 70 | 82 | 56 | 48 | 68 | 73 |
| Y | 76 | 73 | 60 | 41 | 78 | 68 | 69 | 86 | 76 | 67 | 78 | 61 | 49 | 45 | 66 | 66 |
| V | 58 | 72 | 67 | 36 | 61 | 62 | 57 | 67 | 64 | 60 | 44 | 52 | 46 | 41 | 67 | 57 |
| C | 52 | 86 | 75 | 40 | 84 | 51 | 55 | 70 | 42 | 56 | 21 | 46 | 55 | 48 |  | 56 |
| W | 49 |  | 41 | 15 | 54 | 34 | 40 | 43 | 49 | 55 | 44 | 43 | 45 | 40 |  | 42 |
| Average  Binding  Position 1 | 64 | 72 | 57 | 34 | 57 | 49 | 51 | 54 | 57 | 56 | 47 | 51 | 51 | 44 | 69 |  |
