# Supplementary material for: A pH Sensitive High-Throughput Assay for miRNA Binding of a Peptide-Aminoglycoside (PA) Library
Source: PLoS One. 2015 Dec 11;10(12):e0144251. doi: 10.1371/journal.pone.0144251 (PMC4699463; doi:10.1371/journal.pone.0144251)
Supplement: S3 Table — (DOCX) [file pone.0144251.s003.docx]

S3 Table. hsa-miR 335 Percent Binding of Neomycin

| Position 2 | Position 1 | | | | | | | | | | | | | | | |
| --- | --- | --- | --- | --- | --- | --- | --- | --- | --- | --- | --- | --- | --- | --- | --- | --- |
|  | *β*A | R | N | D | H | L | F | P | S | T | Y | V | C | W | K | Average  Binding  Position 2 |
| N/A | 72 | 94 | 80 | 38 | 68 | 65 | 69 | 20 | 78 | 75 | 68 | 51 | 72 | 73 | 81 | 67 |
| βA | 84 | 87 | 62 | 66 | 72 | 30 | 49 | 44 | 73 | 52 | 73 | 45 | 75 | 56 |  | 62 |
| R | 72 | 76 | 65 | 43 | 55 | 65 | 57 | 55 | 75 | 81 | 83 | 68 | 60 | 66 |  | 66 |
| N | 50 | 80 | 61 | 12 | 88 | 24 | 54 | 45 | 58 | 54 | 36 | 31 | 24 | 44 |  | 47 |
| D | 23 | 59 | 39 | 42 | 27 | 18 | 13 | 15 | 6 | 14 | 27 | 20 | 21 | 14 |  | 24 |
| H | 48 | 50 | 65 | 42 | 56 | 40 | 46 | 50 | 43 | 43 | 41 | 41 | 41 | 41 |  | 46 |
| L | 43 | 54 | 37 | 0 | 45 | 5 | 22 | 49 | 40 | 47 | 35 | 26 | 18 | 8 |  | 31 |
| F | 38 | 40 | 27 | 25 | 52 | 35 | 43 | 43 | 50 | 34 | 31 | 31 | 26 | 0 |  | 34 |
| P | 44 | 67 | 48 | 23 | 59 | 38 | 54 | 45 | 42 | 34 | 28 | 29 | 57 | 38 |  | 43 |
| S | 60 | 69 | 64 | 33 | 61 | 56 | 44 | 43 | 57 | 43 | 45 | 70 | 72 | 37 | 76 | 55 |
| T | 69 | 68 | 42 | 29 | 54 | 51 | 64 | 81 | 87 | 88 | 69 | 73 | 44 | 51 | 70 | 63 |
| Y | 76 | 73 | 60 | 41 | 78 | 68 | 69 | 86 | 76 | 67 | 78 | 61 | 49 | 45 | 70 | 66 |
| V | 58 | 72 | 67 | 36 | 61 | 62 | 57 | 67 | 64 | 60 | 44 | 52 | 46 | 41 | 70 | 57 |
| C | 28 | 99 | 80 | 49 | 81 | 51 | 47 | 53 | 49 | 65 | 66 | 43 | 65 | 51 |  | 59 |
| W | 52 |  | 50 | 13 | 53 | 28 | 42 | 39 | 45 | 47 | 42 | 49 | 52 | 43 |  | 43 |
| Average  Binding  Position 1 | 54 | 71 | 56 | 33 | 61 | 42 | 49 | 49 | 56 | 54 | 51 | 46 | 48 | 41 | 73 |  |
