# Supplementary material for: A pH Sensitive High-Throughput Assay for miRNA Binding of a Peptide-Aminoglycoside (PA) Library
Source: PLoS One. 2015 Dec 11;10(12):e0144251. doi: 10.1371/journal.pone.0144251 (PMC4699463; doi:10.1371/journal.pone.0144251)
Supplement: S4 Table — (DOCX) [file pone.0144251.s004.docx]

S4 Table. hsa-miR 504 Percent Binding of Neomycin

| Position 2 | Position 1 | | | | | | | | | | | | | | | |
| --- | --- | --- | --- | --- | --- | --- | --- | --- | --- | --- | --- | --- | --- | --- | --- | --- |
|  | *β*A | R | N | D | H | L | F | P | S | T | Y | V | C | W | K | Average  Binding  Position 2 |
| N/A | 80 | 114 | 87 | 55 | 95 | 82 | 55 | 95 | 82 | 67 | 76 | 62 | 64 | 67 | 86 | 78 |
| βA | 78 | 74 | 71 | 41 | 61 | 48 | 48 | 93 | 93 | 89 | 35 | 39 | 41 | 60 |  | 62 |
| R | 79 | 85 | 93 | 79 | 90 | 78 | 78 | 78 | 89 | 73 | 77 | 89 | 62 | 70 |  | 80 |
| N | 87 | 80 | 63 | 25 | 61 | 73 | 63 | 78 | 65 | 77 | 61 | 59 | 35 | 65 |  | 64 |
| D | 31 | 60 | 26 | 23 | 9 | 13 | 12 | 48 | 64 | 34 | 68 | 56 | 26 | 49 |  | 37 |
| H | 86 | 121 | 114 | 93 | 75 | 104 | 88 | 94 | 103 | 90 | 79 | 84 | 72 | 60 |  | 90 |
| L | 46 | 62 | 98 | 36 | 89 | 79 | 95 | 94 | 57 | 96 | 42 | 87 | 47 | 78 |  | 72 |
| F | 37 | 82 | 83 | 46 | 81 | 71 | 73 | 64 | 78 | 69 | 74 | 76 | 51 | 67 |  | 68 |
| P | 74 | 83 | 79 | 31 | 63 | 50 | 53 | 88 | 69 | 72 | 98 | 117 | 60 | 64 |  | 72 |
| S | 122 | 87 | 127 | 93 | 99 | 107 | 115 | 105 | 106 | 102 | 104 | 107 | 84 | 62 | 70 | 99 |
| T | 86 | 92 | 91 | 59 | 94 | 79 | 94 | 92 | 90 | 76 | 65 | 58 | 25 | 74 | 61 | 76 |
| Y | 76 | 100 | 74 | 41 | 110 | 58 | 106 | 50 | 99 | 98 | 93 | 87 | 47 | 73 | 56 | 78 |
| V | 106 | 106 | 87 | 27 | 87 | 94 | 92 | 86 | 95 | 91 | 83 | 82 | 30 | 51 | 55 | 78 |
| C | 20 | 65 | 61 | 33 | 81 | 55 | 52 | 64 | 68 | 62 | 65 | 45 | 74 | 50 |  | 57 |
| W | 71 | 84 | 74 | 36 | 66 | 57 | 75 | 58 | 71 | 68 | 60 | 63 | 50 | 58 |  | 64 |
| Average  Binding  Position 1 | 72 | 86 | 82 | 48 | 77 | 70 | 73 | 79 | 82 | 78 | 72 | 74 | 51 | 63 | 66 |  |
