# Supplementary material for: A pH Sensitive High-Throughput Assay for miRNA Binding of a Peptide-Aminoglycoside (PA) Library
Source: PLoS One. 2015 Dec 11;10(12):e0144251. doi: 10.1371/journal.pone.0144251 (PMC4699463; doi:10.1371/journal.pone.0144251)
Supplement: S5 Table — (DOCX) [file pone.0144251.s005.docx]

S5 Table. Pre-hsa-miR 504 Percent Binding of Neomycin

| Position 2 | Position 1 | | | | | | | | | | | | | | | |
| --- | --- | --- | --- | --- | --- | --- | --- | --- | --- | --- | --- | --- | --- | --- | --- | --- |
|  | *β*A | R | N | D | H | L | F | P | S | T | Y | V | C | W | K | Average  Binding  Position 2 |
| N/A | 82 | 101 | 89 | 45 | 74 | 77 | 72 | 76 | 73 | 69 | 30 | 27 | 44 | 54 | 70 | 66 |
| βA | 63 | 95 | 61 | 31 | 72 | 69 | 60 | 65 | 56 | 57 | 51 | 53 | 44 | 53 |  | 59 |
| R | 75 | 89 | 74 | 47 | 71 | 28 | 33 | 48 | 56 | 59 | 66 | 71 | 67 | 65 |  | 61 |
| N | 55 | 66 | 48 | 70 | 82 | 63 | 79 | 87 | 79 | 88 | 81 | 79 | 59 | 80 |  | 73 |
| D | 63 | 84 | 68 | 74 | 37 | 38 | 48 | 40 | 55 | 54 | 43 | 42 | 69 | 34 |  | 54 |
| H | 66 | 102 | 63 | 53 | 75 | 44 | 42 | 22 | 71 | 95 | 82 | 75 | 71 | 70 |  | 67 |
| L | 67 | 84 | 60 | 28 | 71 | 46 | 50 | 41 | 64 | 64 | 59 | 31 | 29 | 52 |  | 53 |
| F | 71 | 88 | 57 | 28 | 74 | 48 | 68 | 55 | 62 | 68 | 59 | 49 | 62 | 40 |  | 59 |
| P | 43 | 51 | 36 | 3 | 107 | 85 | 104 | 84 | 98 | 95 | 74 | 85 | 83 | 91 |  | 74 |
| S | 93 | 118 | 97 | 71 | 94 | 92 | 93 | 74 | 94 | 72 | 78 | 89 | 71 | 58 | 83 | 85 |
| T | 62 | 63 | 92 | 38 | 86 | 92 | 93 | 87 | 88 | 78 | 84 | 81 | 58 | 68 | 74 | 76 |
| Y | 84 | 106 | 88 | 47 | 94 | 90 | 92 | 83 | 76 | 63 | 66 | 47 | 25 | 90 | 74 | 75 |
| V | 73 | 112 | 92 | 61 | 98 | 86 | 97 | 89 | 85 | 86 | 86 | 78 | 71 | 67 | 62 | 83 |
| C | 52 | 91 | 60 | 37 | 63 | 43 | 33 | 46 | 59 | 58 | 79 | 74 | 52 | 68 |  | 58 |
| W | 59 | 75 | 73 | 40 | 78 | 61 | 70 | 67 | 71 | 66 | 63 | 67 | 57 | 62 |  | 65 |
| Average  Binding  Position 1 | 67 | 88 | 71 | 45 | 78 | 64 | 69 | 64 | 72 | 71 | 67 | 63 | 57 | 63 | 73 |  |
