# Supplementary material for: A pH Sensitive High-Throughput Assay for miRNA Binding of a Peptide-Aminoglycoside (PA) Library
Source: PLoS One. 2015 Dec 11;10(12):e0144251. doi: 10.1371/journal.pone.0144251 (PMC4699463; doi:10.1371/journal.pone.0144251)
Supplement: S6 Table — (DOCX) [file pone.0144251.s006.docx]

S6 Table. hsa-miR 142 Standard deviation from the mean of all compounds (σ)

| Position 2 | Position 1 | | | | | | | | | | | | | | | |
| --- | --- | --- | --- | --- | --- | --- | --- | --- | --- | --- | --- | --- | --- | --- | --- | --- |
|  | *β*A | R | N | D | H | L | F | P | S | T | Y | V | C | W | K | Average  σ  Position 2 |
| N/A | 0.12 | 2.04 | 0.60 | -0.64 | -0.31 | -0.74 | -0.84 | -1.65 | -1.94 | -0.79 | -0.40 | -2.04 | -0.74 | -0.31 | 1.13 | -0.43 |
| βA | 1.32 | 0.27 | -1.84 | -1.84 | -1.27 | -0.07 | 0.07 | -0.02 | -1.12 | -1.51 | -1.17 | -0.12 | 0.55 | -1.22 |  | -0.57 |
| R | 1.03 | 0.84 | 1.18 | -0.84 | 0.75 | -0.36 | -0.55 | -1.22 | 0.94 | -0.84 | 0.17 | 0.07 | 0.41 | 0.17 |  | 0.13 |
| N | 0.55 | -0.45 | -1.27 | -2.13 | -1.03 | 0.31 | 0.27 | 0.65 | 0.84 | 0.60 | -1.84 | -1.36 | -1.46 | -0.21 |  | -0.47 |
| D | -0.55 | 0.99 | -1.22 | -0.12 | -0.79 | -1.84 | -1.70 | -1.80 | -1.41 | -1.65 | -1.99 | -1.60 | -1.56 | -1.84 |  | -1.22 |
| H | 0.07 | 0.31 | -0.12 | -1.46 | -0.31 | -1.12 | -0.88 | -1.12 | 0.07 | 0.55 | -0.93 | -0.64 | -1.32 | -0.84 |  | -0.55 |
| L | -0.64 | 0.07 | -0.69 | -2.47 | -0.88 | -1.22 | -1.03 | 0.12 | 0.65 | 0.99 | 0.94 | 0.60 | 0.51 | -0.74 |  | -0.27 |
| F | 0.60 | 0.70 | 1.18 | -0.21 | 0.99 | 0.36 | 0.36 | 0.12 | 0.65 | 0.55 | -0.02 | 0.22 | -0.45 | -0.55 |  | 0.32 |
| P | -0.21 | 1.32 | 0.99 | -0.16 | 0.99 | 0.22 | 0.55 | 0.46 | 0.70 | -0.16 | 0.31 | -0.02 | -0.21 | -0.26 |  | 0.32 |
| S | 1.66 | 1.75 | 1.61 | 0.03 | 1.08 | 0.79 | 0.51 | 0.17 | 0.65 | 0.79 | 1.03 | 2.62 | 3.43 | 1.51 | 0.55 | 1.21 |
| T | 2.42 | 0.89 | 0.65 | 0.03 | 0.07 | 0.36 | 1.42 | 1.95 | 1.90 | 1.80 | 0.79 | 1.37 | 0.12 | -0.26 | 0.70 | 0.95 |
| Y | 1.08 | 0.94 | 0.31 | -0.60 | 1.18 | 0.70 | 0.75 | 1.56 | 1.08 | 0.65 | 1.18 | 0.36 | -0.21 | -0.40 | 0.60 | 0.61 |
| V | 0.22 | 0.89 | 0.65 | -0.84 | 0.36 | 0.41 | 0.17 | 0.65 | 0.51 | 0.31 | -0.45 | -0.07 | -0.36 | -0.60 | 0.65 | 0.17 |
| C | -0.07 | 1.56 | 1.03 | -0.64 | 1.47 | -0.12 | 0.07 | 0.79 | -0.55 | 0.12 | -1.56 | -0.36 | 0.07 | -0.26 |  | 0.11 |
| W | -0.21 | -2.56 | -0.60 | -1.84 | 0.03 | -0.93 | -0.64 | -0.50 | -0.21 | 0.07 | -0.45 | -0.50 | -0.40 | -0.64 |  | -0.67 |
| Average  σ  Position 1 | 0.49 | 0.64 | 0.16 | -0.92 | 0.15 | -0.22 | -0.10 | 0.01 | 0.18 | 0.10 | -0.29 | -0.10 | -0.11 | -0.43 | 0.73 |  |
