# Supplementary material for: A pH Sensitive High-Throughput Assay for miRNA Binding of a Peptide-Aminoglycoside (PA) Library
Source: PLoS One. 2015 Dec 11;10(12):e0144251. doi: 10.1371/journal.pone.0144251 (PMC4699463; doi:10.1371/journal.pone.0144251)
Supplement: S7 Table — (DOCX) [file pone.0144251.s007.docx]

S7 Table**.** hsa-miR 335 Standard deviation from the mean of all compounds (σ)

| Position 2 | Position 1 | | | | | | | | | | | | | | | |
| --- | --- | --- | --- | --- | --- | --- | --- | --- | --- | --- | --- | --- | --- | --- | --- | --- |
|  | *β*A | R | N | D | H | L | F | P | S | T | Y | V | C | W | K | Average  σ  Position 2 |
| N/A | 1.06 | 2.18 | 1.47 | -0.67 | 0.86 | 0.70 | 0.91 | -1.59 | 1.36 | 1.21 | 0.86 | -0.01 | 1.06 | 1.11 | 1.52 | 0.80 |
| βA | 1.67 | 1.82 | 0.55 | 0.75 | 1.06 | -1.08 | -0.11 | -0.37 | 1.11 | 0.04 | 1.11 | -0.32 | 1.21 | 0.24 |  | 0.55 |
| R | 1.06 | 1.26 | 0.70 | -0.42 | 0.19 | 0.70 | 0.30 | 0.19 | 1.21 | 1.52 | 1.62 | 0.86 | 0.45 | 0.75 |  | 0.74 |
| N | -0.06 | 1.47 | 0.50 | -2.00 | 1.87 | -1.38 | 0.14 | -0.32 | 0.35 | 0.14 | -0.77 | -1.03 | -1.38 | -0.37 |  | -0.20 |
| D | -1.44 | 0.40 | -0.62 | -0.47 | -1.23 | -1.69 | -1.94 | -1.84 | -2.30 | -1.89 | -1.23 | -1.59 | -1.54 | -1.89 |  | -1.38 |
| H | -0.16 | -0.06 | 0.70 | -0.47 | 0.24 | -0.57 | -0.26 | -0.06 | -0.42 | -0.42 | -0.52 | -0.52 | -0.52 | -0.52 |  | -0.25 |
| L | -0.42 | 0.14 | -0.72 | -2.61 | -0.32 | -2.35 | -1.49 | -0.11 | -0.57 | -0.21 | -0.82 | -1.28 | -1.69 | -2.20 |  | -1.05 |
| F | -0.67 | -0.57 | -1.23 | -1.33 | 0.04 | -0.82 | -0.42 | -0.42 | -0.06 | -0.88 | -1.03 | -1.03 | -1.28 | -2.61 |  | -0.88 |
| P | -0.37 | 0.80 | -0.16 | -1.44 | 0.40 | -0.67 | 0.14 | -0.32 | -0.47 | -0.88 | -1.18 | -1.13 | 0.30 | -0.67 |  | -0.40 |
| S | 0.45 | 0.91 | 0.65 | -0.93 | 0.50 | 0.24 | -0.37 | -0.42 | 0.30 | -0.42 | -0.32 | 0.96 | 1.06 | -0.72 | 1.26 | 0.21 |
| T | 0.91 | 0.86 | -0.47 | -1.13 | 0.14 | -0.01 | 0.65 | 1.52 | 1.82 | 1.87 | 0.91 | 1.11 | -0.37 | -0.01 | 0.96 | 0.58 |
| Y | 1.26 | 1.11 | 0.45 | -0.52 | 1.36 | 0.86 | 0.91 | 1.77 | 1.26 | 0.80 | 1.36 | 0.50 | -0.11 | -0.32 | 0.96 | 0.78 |
| V | 0.35 | 1.06 | 0.80 | -0.77 | 0.50 | 0.55 | 0.30 | 0.80 | 0.65 | 0.45 | -0.37 | 0.04 | -0.26 | -0.52 | 0.96 | 0.30 |
| C | -1.18 | 2.43 | 1.47 | -0.11 | 1.52 | -0.01 | -0.21 | 0.09 | -0.11 | 0.70 | 0.75 | -0.42 | 0.70 | -0.01 |  | 0.40 |
| W | 0.04 | -2.61 | -0.06 | -1.94 | 0.09 | -1.18 | -0.47 | -0.62 | -0.32 | -0.21 | -0.47 | -0.11 | 0.04 | -0.42 |  | -0.59 |
| Average  σ  Position 1 | 0.17 | 0.75 | 0.27 | -0.94 | 0.48 | -0.45 | -0.13 | -0.11 | 0.26 | 0.12 | -0.01 | -0.26 | -0.16 | -0.54 | 1.13 |  |
