# Supplementary material for: A pH Sensitive High-Throughput Assay for miRNA Binding of a Peptide-Aminoglycoside (PA) Library
Source: PLoS One. 2015 Dec 11;10(12):e0144251. doi: 10.1371/journal.pone.0144251 (PMC4699463; doi:10.1371/journal.pone.0144251)
Supplement: S8 Table — (DOCX) [file pone.0144251.s008.docx]

S8 Table. hsa-miR 504 Standard deviation from the mean of all compounds (σ)

| Position 2 | Position 1 | | | | | | | | | | | | | | | |
| --- | --- | --- | --- | --- | --- | --- | --- | --- | --- | --- | --- | --- | --- | --- | --- | --- |
|  | *β*A | R | N | D | H | L | F | P | S | T | Y | V | C | W | K | Average  σ  Position 2 |
| N/A | 0.36 | 1.84 | 0.66 | -0.74 | 1.01 | 0.45 | -0.74 | 1.01 | 0.45 | -0.21 | 0.18 | -0.43 | -0.34 | -0.21 | 0.62 | 0.26 |
| βA | 0.27 | 0.10 | -0.04 | -1.35 | -0.47 | -1.04 | -1.04 | 0.93 | 0.93 | 0.75 | -1.61 | -1.44 | -1.35 | -0.52 |  | -0.42 |
| R | 0.31 | 0.58 | 0.93 | 0.31 | 0.80 | 0.27 | 0.27 | 0.27 | 0.75 | 0.05 | 0.23 | 0.75 | -0.43 | -0.08 |  | 0.36 |
| N | 0.66 | 0.36 | -0.39 | -2.05 | -0.47 | 0.05 | -0.39 | 0.27 | -0.30 | 0.23 | -0.47 | -0.56 | -1.61 | -0.30 |  | -0.35 |
| D | -1.79 | -0.52 | -2.00 | -2.14 | -2.75 | -2.57 | -2.62 | -1.04 | -0.34 | -1.65 | -0.17 | -0.69 | -2.00 | -1.00 |  | -1.52 |
| H | 0.62 | 2.15 | 1.84 | 0.93 | 0.14 | 1.41 | 0.71 | 0.97 | 1.36 | 0.80 | 0.31 | 0.53 | 0.01 | -0.52 |  | 0.80 |
| L | -1.13 | -0.43 | 1.15 | -1.57 | 0.75 | 0.31 | 1.01 | 0.97 | -0.65 | 1.06 | -1.30 | 0.66 | -1.09 | 0.27 |  | 0.00 |
| F | -1.52 | 0.45 | 0.49 | -1.13 | 0.40 | -0.04 | 0.05 | -0.34 | 0.27 | -0.12 | 0.10 | 0.18 | -0.91 | -0.21 |  | -0.17 |
| P | 0.10 | 0.49 | 0.31 | -1.79 | -0.39 | -0.95 | -0.82 | 0.71 | -0.12 | 0.01 | 1.15 | 1.98 | -0.52 | -0.34 |  | -0.01 |
| S | 2.19 | 0.66 | 2.41 | 0.93 | 1.19 | 1.54 | 1.89 | 1.45 | 1.49 | 1.32 | 1.41 | 1.54 | 0.53 | -0.43 | -0.08 | 1.20 |
| T | 0.62 | 0.88 | 0.84 | -0.56 | 0.97 | 0.31 | 0.97 | 0.88 | 0.80 | 0.18 | -0.30 | -0.60 | -2.05 | 0.10 | -0.47 | 0.17 |
| Y | 0.18 | 1.23 | 0.10 | -1.35 | 1.67 | -0.60 | 1.49 | -0.95 | 1.19 | 1.15 | 0.93 | 0.66 | -1.09 | 0.05 | -0.69 | 0.26 |
| V | 1.49 | 1.49 | 0.66 | -1.96 | 0.66 | 0.97 | 0.88 | 0.62 | 1.01 | 0.84 | 0.49 | 0.45 | -1.83 | -0.91 | -0.74 | 0.28 |
| C | -2.27 | -0.30 | -0.47 | -1.70 | 0.40 | -0.74 | -0.87 | -0.34 | -0.17 | -0.43 | -0.30 | -1.17 | 0.10 | -0.95 |  | -0.66 |
| W | -0.04 | 0.53 | 0.10 | -1.57 | -0.25 | -0.65 | 0.14 | -0.60 | -0.04 | -0.17 | -0.52 | -0.39 | -0.95 | -0.60 |  | -0.36 |
| Average  σ  Position 1 | 0.00 | 0.63 | 0.44 | -1.05 | 0.24 | -0.09 | 0.06 | 0.32 | 0.44 | 0.25 | 0.01 | 0.10 | -0.90 | -0.38 | -0.27 |  |
