# Supplementary material for: A pH Sensitive High-Throughput Assay for miRNA Binding of a Peptide-Aminoglycoside (PA) Library
Source: PLoS One. 2015 Dec 11;10(12):e0144251. doi: 10.1371/journal.pone.0144251 (PMC4699463; doi:10.1371/journal.pone.0144251)
Supplement: S9 Table — (DOCX) [file pone.0144251.s009.docx]

S9 Table. Pre-hsa-miR 504 Standard deviation from the mean of all compounds (σ)

| Position 2 | Position 1 | | | | | | | | | | | | | | | |
| --- | --- | --- | --- | --- | --- | --- | --- | --- | --- | --- | --- | --- | --- | --- | --- | --- |
|  | *β*A | R | N | D | H | L | F | P | S | T | Y | V | C | W | K | Average  σ  Position 2 |
| N/A | 0.74 | 1.70 | 1.10 | -1.13 | 0.34 | 0.49 | 0.23 | 0.44 | 0.28 | 0.08 | -1.89 | -2.04 | -1.18 | -0.68 | 0.13 | -0.09 |
| βA | -0.22 | 1.40 | -0.32 | -1.84 | 0.23 | 0.08 | -0.37 | -0.12 | -0.58 | -0.53 | -0.83 | -0.73 | -1.18 | -0.73 |  | -0.41 |
| R | 0.39 | 1.10 | 0.34 | -1.03 | 0.18 | -1.99 | -1.74 | -0.98 | -0.58 | -0.42 | -0.07 | 0.18 | -0.02 | -0.12 |  | -0.34 |
| N | -0.63 | -0.07 | -0.98 | 0.13 | 0.74 | -0.22 | 0.59 | 0.99 | 0.59 | 1.04 | 0.69 | 0.59 | -0.42 | 0.64 |  | 0.26 |
| D | -0.22 | 0.84 | 0.03 | 0.34 | -1.54 | -1.49 | -0.98 | -1.39 | -0.63 | -0.68 | -1.23 | -1.28 | 0.08 | -1.69 |  | -0.70 |
| H | -0.07 | 1.75 | -0.22 | -0.73 | 0.39 | -1.18 | -1.28 | -2.30 | 0.18 | 1.40 | 0.74 | 0.39 | 0.18 | 0.13 |  | -0.04 |
| L | -0.02 | 0.84 | -0.37 | -1.99 | 0.18 | -1.08 | -0.88 | -1.34 | -0.17 | -0.17 | -0.42 | -1.84 | -1.94 | -0.78 |  | -0.71 |
| F | 0.18 | 1.04 | -0.53 | -1.99 | 0.34 | -0.98 | 0.03 | -0.63 | -0.27 | 0.03 | -0.42 | -0.93 | -0.27 | -1.39 |  | -0.41 |
| P | -1.23 | -0.83 | -1.59 | -3.26 | 2.01 | 0.89 | 1.85 | 0.84 | 1.55 | 1.40 | 0.34 | 0.89 | 0.79 | 1.20 |  | 0.35 |
| S | 1.30 | 2.56 | 1.50 | 0.18 | 1.35 | 1.25 | 1.30 | 0.34 | 1.35 | 0.23 | 0.54 | 1.10 | 0.18 | -0.47 | 0.79 | 0.90 |
| T | -0.27 | -0.22 | 1.25 | -1.49 | 0.94 | 1.25 | 1.30 | 0.99 | 1.04 | 0.54 | 0.84 | 0.69 | -0.47 | 0.03 | 0.34 | 0.45 |
| Y | 0.84 | 1.96 | 1.04 | -1.03 | 1.35 | 1.15 | 1.25 | 0.79 | 0.44 | -0.22 | -0.07 | -1.03 | -2.15 | 1.15 | 0.34 | 0.39 |
| V | 0.28 | 2.26 | 1.25 | -0.32 | 1.55 | 0.94 | 1.50 | 1.10 | 0.89 | 0.94 | 0.94 | 0.54 | 0.18 | -0.02 | -0.27 | 0.78 |
| C | -0.78 | 1.20 | -0.37 | -1.54 | -0.22 | -1.23 | -1.74 | -1.08 | -0.42 | -0.47 | 0.59 | 0.34 | -0.78 | 0.03 |  | -0.46 |
| W | -0.42 | 0.39 | 0.28 | -1.39 | 0.54 | -0.32 | 0.13 | -0.02 | 0.18 | -0.07 | -0.22 | -0.02 | -0.53 | -0.27 |  | -0.12 |
| Average  σ  Position 1 | -0.01 | 1.06 | 0.16 | -1.14 | 0.56 | -0.16 | 0.08 | -0.16 | 0.26 | 0.21 | -0.03 | -0.21 | -0.50 | -0.20 | 0.26 |  |
